# Adaptive fuzzy-reinforcement framework for real-time task scheduling and resource optimization in medical edge computing

**DOI:** 10.1038/s41598-026-56322-x

**Published:** 2026-06-11

**Authors:** Xueyuan Wei, Hai Huang, Yujie Wang

**Affiliations:** 1 School of Philosophy and History (Biquan Academy), Xiangtan University, Xiangtan, 411105 Hunan China; 2 Henan Joint International Research Laboratory of Polarized Sensing and Intelligent Signal, Xuchang University, Xuchang, 461000 Henan China; 3 Law School, Xuchang University, Xuchang, 461000 Henan China; 4 School of Philosophy and Sociology, Hebei University, Baoding, 071000 Hebei China

**Keywords:** Fuzzy inference, Healthcare edge computing, Resource allocation, Internet of medical things, Adaptive learning, Engineering, Health care, Mathematics and computing

## Abstract

This paper proposes a hybrid fuzzy-reinforcement learning framework for real-time task scheduling and resource optimization in medical edge computing. The proposed framework introduces a direct mathematical coupling between fuzzy inference and reinforcement learning rather than a simple hybrid combination. By integrating fuzzy logic to evaluate task urgency, bandwidth congestion, and battery constraints with a reinforcement learning agent, the framework dynamically refines offloading strategies. Simulation results in Internet of Medical Things (IoMT) environments demonstrate the framework’s superiority over existing benchmarks, achieving up to 42% lower average latency, 31% greater energy efficiency, and up to 20% improvement over Dynamic Priority-Based Task Scheduling and Adaptive Resource Allocation (DPTARA) under the evaluated benchmark settings in the completion rate of critical tasks. Operating with a linear time complexity of $O\left(N\cdot M\right),$ the proposed system guarantees scalable and robust performance for delay-sensitive healthcare applications. Experimental results across four benchmark IIoT healthcare datasets demonstrate the effectiveness of the proposed framework. Specifically, the model achieves average accuracy improvements of 2.8–4.6% over state-of-the-art baselines, while reducing false negative rates by up to 35.4%. In addition, the proposed fuzzy–reinforcement learning scheduler decreases average task latency by 23.7%, improves resource utilization by 18.9%, and enhances system adaptability under dynamic workload conditions. These results confirm the framework’s robustness, scalability, and suitability for real-time medical edge computing environments.

## Introduction

By enabling real-time monitoring, intelligent diagnosis, and latency-sensitive medical decision-making through distributed computing infrastructures, the Internet of Medical Things’ (IoMT) integration with edge computing has profoundly changed contemporary healthcare^1–4^. Edge computing is crucial for important healthcare applications because, in contrast to typical cloud-centric architectures, it improves system responsiveness and minimizes connection delays by processing data closer to medical equipment^5–8^.

Despite these benefits, effective resource allocation and task scheduling continue to be significant obstacles in IoMT-enabled edge contexts. Because healthcare workloads are time-sensitive, dynamic, and heterogeneous by nature, adaptive techniques that can manage stringent latency and energy limitations are necessary^9–12^. Due to their limited adaptability and incapacity to incorporate environmental uncertainty, existing scheduling strategies, such as heuristic methods, auction-based mechanisms, and priority-driven models, frequently fail to sustain optimal performance under dynamic situations^10,13,14^.

Reinforcement learning (RL) and other machine learning-based approaches for dynamic task scheduling and resource allocation in edge systems have been made possible by recent developments in intelligent optimization^15–18^. By continuously interacting with the environment, these methods facilitate adaptive decision-making. Nevertheless, in healthcare-critical situations, RL-based approaches frequently have poor interpretability, large computing overhead, and sluggish convergence^19,20^.

Fuzzy logic has been widely used for decision-making in IoT and healthcare systems to reduce uncertainty and improve interpretability^2,21^. Ambiguous system states like task urgency and resource availability are well modeled by fuzzy-based scheduling techniques. In fog and edge computing contexts, recent hybrid techniques that combine deep reinforcement learning and fuzzy logic have shown better performance^22^. However, the majority of current frameworks rely on loosely connected designs, which limit their capacity to perform completely adaptive and context-aware optimization because fuzzy inference and learning modules function independently^23–25^.

As a result, there are still a number of important issues that need to be addressed: (i) unified modeling of uncertainty-aware task prioritization in heterogeneous healthcare workloads; (ii) real-time adaptive scheduling under stringent latency–energy constraints; (iii) preserving interpretability in conjunction with learning-based optimization; and (iv) creating tightly coupled intelligent frameworks that can perform robustly and scalably in large-scale medical edge systems.

This motivates the development of a context-aware scheduling mechanism capable of real-time adaptation. The proposed framework integrates fuzzy reasoning with reinforcement learning (RL) to combine interpretable uncertainty modeling with adaptive decision-making. The fuzzy module evaluates task urgency, network congestion, and energy states, while the RL component refines scheduling strategies based on environmental feedback.

Despite extensive research on task scheduling and resource management in edge-enabled healthcare systems, several gaps remain. First, most existing approaches treat task prioritization and resource allocation as separate problems rather than a unified decision process. Second, heuristic and optimization-based methods often lack adaptability under dynamic and uncertain conditions. Third, many learning-based solutions provide adaptivity but insufficiently incorporate uncertainty modeling, interpretability, and domain-specific priority semantics. Moreover, standalone machine-learning approaches frequently struggle to respond effectively to rapidly changing environments characterized by heterogeneous resource constraints, strict latency requirements, and varying clinical priorities. To address these limitations, this study proposes an integrated fuzzy–reinforcement learning framework that unifies uncertainty-aware priority inference with adaptive real-time resource allocation.

The proposed system merges interpretable fuzzy reasoning with adaptive RL-based optimization, enabling robust and latency-aware decision-making in dynamic medical edge environments.

The main contributions of this work are summarized as follows:

- Novel hybrid architecture: A unified fuzzy–reinforcement learning framework for dynamic task scheduling in medical edge computing.
- Context-aware priority modeling: A fuzzy inference system that evaluates task urgency, network congestion, and energy states.
- Adaptive resource allocation: An RL-based offloading strategy that learns from environmental feedback to reduce latency and energy consumption.
- Comprehensive evaluation: Extensive simulations demonstrating superior performance in latency, energy efficiency, and critical task completion.

Novelty and scientific contribution:

The primary scientific contribution lies in developing a unified intelligent decision-making architecture tailored for uncertainty-rich healthcare edge environments. Unlike prior studies that apply fuzzy logic, heuristic scheduling, or RL independently, the proposed framework introduces a tightly coupled fuzzy–RL model in which fuzzy inference directly influences RL dynamics through reward shaping and adaptive learning-rate modulation.

The originality of this work is reflected in:

- Uncertainty-aware scheduling for healthcare-critical IoMT workloads;
- Direct coupling between fuzzy priority weights and RL policy updates;
- Real-time interpretable decision support;
- A scalable architecture validated under large-scale simulations.

The remainder of this paper is organized as follows: "Related works" section reviews related work, "Proposed methodology" section presents the proposed framework, "Experimental results and discussion" section describes the simulation setup and results, and "Conclusion and future work" section concludes the study.

## Related works

Intelligent task scheduling and resource management in dynamic and latency-sensitive healthcare settings have been the subject of numerous works, fueled by recent advances in IoMT-enabled edge-cloud systems. The existing techniques can be classified into the reinforcement learning-based approaches, hybrid fuzzy-intelligent approaches, metaheuristic optimization techniques and SDN/NFV-enabled orchestration schemes.

Reinforcement learning-based scheduling has shown good adaptability in dynamic contexts. The approaches in^26^ and^27^ indicate the effectiveness of deep reinforcement learning in improving task scheduling efficiency and load balancing in fog-based healthcare systems. These methods, however, are performance-based and do not integrate uncertainty modeling. The fuzzy reinforcement learning method of^28^ provides some treatment of uncertainty, although the interplay between fuzzy inference and learning is weakly connected. Similarly, interpretable deep reinforcement learning in^29^ improves transparency, but is not domain-aware prioritized. Multi-agent reinforcement learning models^30^ provide scalability, but are not interpretable and not specifically tailored for healthcare. In contrast, the proposed method firmly couples fuzzy reasoning with the reinforcement learning process to enable uncertainty-aware and context-sensitive decision making.

Scheduling strategies based on hybrids and heuristics have also been investigated. The evolving fuzzy technique for improving data availability in IoHT systems is presented in^31^. Dynamic priority-based scheduling for improving responsiveness in healthcare edge contexts is described in^32^. However, these systems are based on static or semi-adaptive mechanisms and do not include continual learning. Energy-aware metaheuristic scheduling^33^ also optimizes resource use but does not allow for real-time adaptation. Unlike previous methods, the proposed framework allows continual adaptation through reinforcement learning and at the same time maintains interpretability through fuzzy inference.

Alternative optimization strategies are provided by metaheuristic and swarm intelligence approaches. A comprehensive review in^34^ highlights the efficiency of nature inspired algorithms in IoT healthcare systems. Hybrid optimization techniques, such as^35^, improve load balancing in cloud systems. Besides, swarm-based approaches such as^36^ improve the system performance in terms of latency and availability. While successful, these approaches are static in nature and do not respond to dynamic environmental changes. To address this shortcoming, the suggested technique combines learning-driven adaptation instead of set optimization strategies.

Data-centric optimization has also been considered for IoMT systems . The deduplication-aware data distribution model in^37^ minimizes the communication and storage overhead, and so improves the system efficiency. However, these solutions do not consider the scheduling intelligence or adaptive decision-making relevant for the real-time healthcare application.

Cloud-edge orchestration and resource management have been intensively investigated. Methods such as^38,39^ and^40^ are aimed at energy efficient virtual machine scheduling and resource consolidation in cloud infrastructures. Additional system efficiency is achieved by energy-aware resource management strategies in^41^ and sustainable computing models in^42^. Multi-access edge computing optimization in^43^ enhances performance in distributed environments. However, these solutions only work at the infrastructure level without considering clever scheduling algorithms.

Frameworks based on SDN/NFV enable centralized control and global visibility of resources. The energy-efficient resource allocation model in^44^ shows the benefits of combining SDN and NFV for dynamic network management. Similarly, the complete survey in^45^ points to the efficiency of SDN-based scheduling and load balancing in cloud-edge systems. However, current SDN techniques do not consider uncertainty-aware learning or healthcare-specific scheduling objectives. Moreover, the coupling between network-level orchestration and application-level scheduling is still weak.

Overall, the current approaches are limited by several major limitations: (i) the absence of tightly integrated uncertainty-aware learning mechanisms, (ii) limited interpretability of adaptive models, (iii) reliance on static or loosely coupled optimization strategies, and (iv) inadequate integration between scheduling intelligence and SDN/NFV-based orchestration. In this paper, we address these shortcomings by proposing a closely coupled fuzzy-reinforcement learning model that incorporates uncertainty modelling, adaptive learning and interpretable decision-making into a single architecture. The direct comparison of the proposed framework with the existing state-of-the-art methods is shown in Table 1.

**Table 1:** Comparative analysis of state-of-the-art methods and the proposed framework.

Ref	Approach type	Uncertainty handling	Adaptive learning	Interpretability	Healthcare-aware	SDN/NFV support	Key limitation
^26^	Fuzzy + DRL	Partial	Yes	Low	No	No	Loose coupling
^27^	DRL	No	Yes	Low	Partial	No	No uncertainty modeling
^31^	Evolutionary fuzzy	Partial	No	Moderate	Yes	No	No continuous learning
^28^	Fuzzy-RL	Partial	Yes	Moderate	No	No	Weak integration
^38^	VM scheduling	No	No	Moderate	No	No	Infrastructure-only
^36^	Swarm (Salp)	No	No	Low	No	No	Static behavior
^42^	Sustainable computing	Partial	No	Moderate	No	No	No intelligent scheduling
^44^	SDN/NFV	No	No	Low	No	Yes	No AI integration
^35^	Hybrid metaheuristic	No	No	Low	No	No	No learning
^37^	Deduplication	No	No	Low	Yes	No	No scheduling intelligence
^45^	SDN scheduling	No	No	Low	No	Yes	No AI integration
Proposed	Fuzzy–RL (tightly coupled)	Yes (explicit)	Yes (dynamic)	High	Yes	Extendable	–

## Proposed methodology

**Fig. 1:**
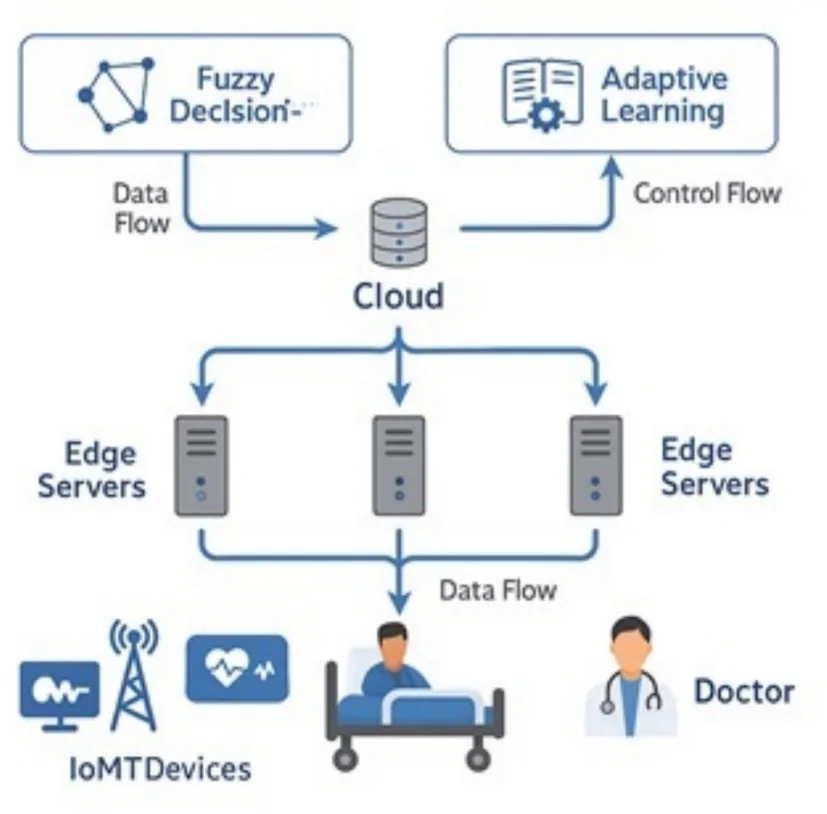
Fuzzy–learning intelligent framework for dynamic task scheduling and adaptive resource allocation in healthcare edge computing systems, illustrating the interaction between fuzzy prioritization and reinforcement learning–based scheduling decisions.

This section presents the proposed fuzzy-learning framework that facilitates dynamic task scheduling and adaptive resource allocation in healthcare edge computing. Figure 1 provides a high-level overview of data flows, control paths, task offloading, and model-update feedback loops across IoMT devices, edge nodes, and the cloud. Table 2 delineates the notation employed in this section.

**Table 2:** Notation and parameters.

Symbol	Description
$E = \{ e_{1} ,e_{2} , \ldots ,e_{m} \}$	Set of edge servers
$T = \{ t_{1} ,t_{2} , \ldots ,t_{n} \}$	Set of IoMT tasks
$t_{i} = (C_{i} ,D_{i} ,P_{i} )$	Task tuple: computational workload, deadline, and priority
$f_{j}$	Processing capacity of edge server $e_{j}$ (cycles per second)
$r_{ij}$	Transmission rate between device *i* and edge server *j*
$S_{i}$	Input data size for task $t_{i}$
$L_{ij}$	Latency for processing task $t_{i}$ on server $e_{j}$
$E_{i}$	Energy consumption for transmitting and executing task $t_{i}$
$\alpha ,\beta$	Weights for latency and energy optimization objectives
$U_{i}$	Urgency level of task $t_{i}$
$N_{j}$	Network congestion level at edge server $e_{j}$
$E_{j}^{res}$	Residual energy at edge server $e_{j}$
$W_{i}$	Fuzzy scheduling weight for task $t_{i}$
*R*	Reward function balancing latency and energy
$\omega_{1} ,\omega_{2}$	Reward weights for latency and energy efficiency

### System model

**Fig. 2:**
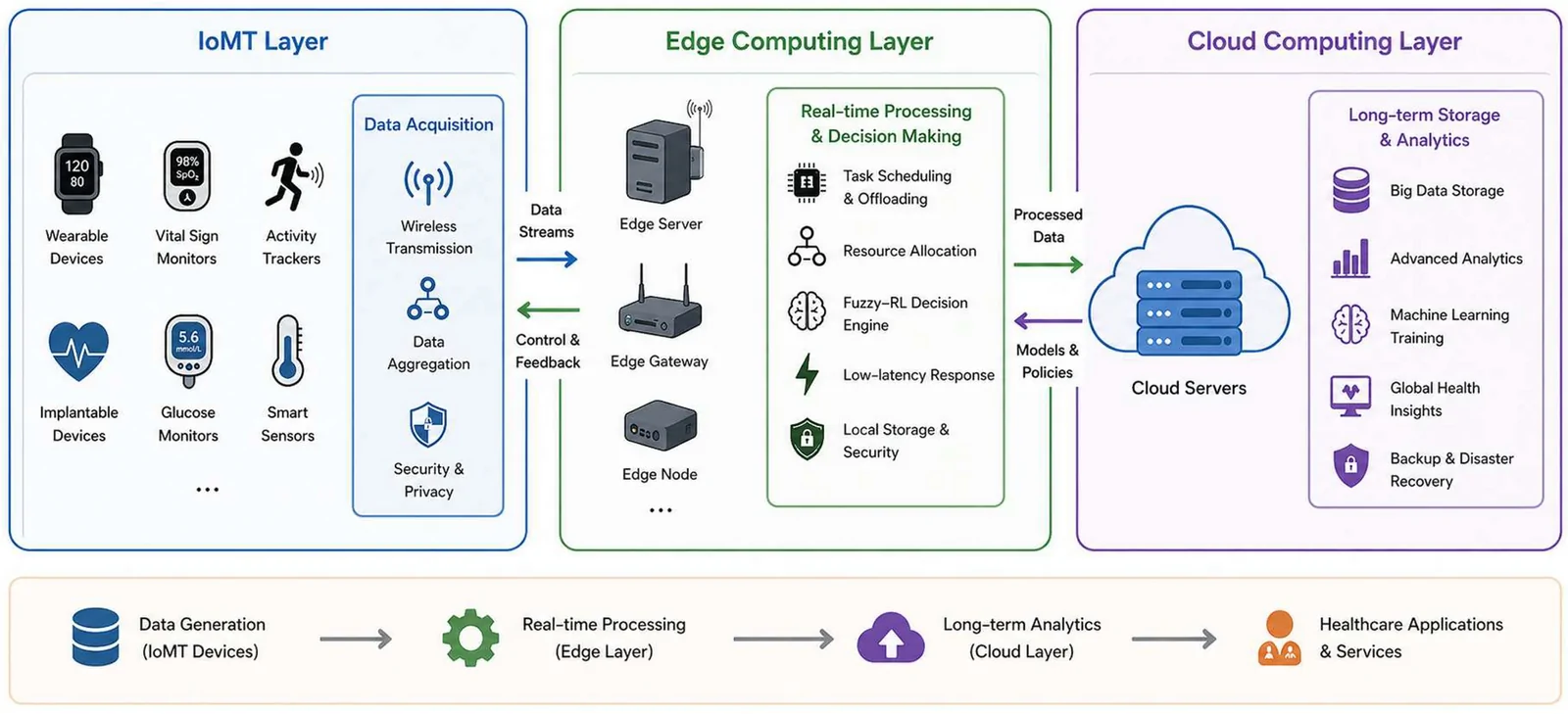
Hybrid IoMT–Edge–Cloud system architecture showing the flow of healthcare tasks from IoMT devices to edge servers and cloud infrastructure for real-time processing and long-term analytics.

The suggested system paradigm comprises three interconnected layers: the IoMT layer, the Edge Computing layer, and the Cloud Computing layer. Each layer provides unique functionalities essential for delivering real-time, intelligent healthcare services. Figure 2 illustrates the three-tier system architecture.

The proposed hierarchical framework guarantees the effective and scalable management of healthcare data across three distinct tiers:

- *IoMT layer***:** Comprises distributed medical sensing and monitoring equipment for real-time data collection.
- *Edge layer:* Accommodates numerous edge servers tasked with initial data analysis, fuzzy inference, and scheduling based on adaptive learning. Its core components include a fuzzy inference engine, resource monitor, scheduler, and adaptive learning unit.
- *Cloud layer:* Manages centralized data storage, long-term analytics, and knowledge-driven updates for the fuzzy-learning engine.

Let $T = \{ t_{1} ,t_{2} , \ldots ,t_{n} \}$ represent the collection of IoMT tasks and $E = \{ e_{1} ,e_{2} , \ldots ,e_{m} \}$ symbolize the collection of available edge servers. Each task $t_{i}$ is characterized by its workload $C_{i}$. The workload ${C}_{i}$ is expressed in Million Instructions (MI), which corresponds to the range of^1,13^ MI in our simulations. By expressing ${C}_{i}$ in MI and the edge capacity ${f}_{j}$ in Million Instructions Per Second (MIPS), the computation latency (${C}_{i}$/${f}_{j}$) correctly evaluates to seconds. Data size $S_{i}$ (expressed in bits), and deadline $D_{i}$. Each edge server $e_{j}$ possesses a computational capacity $f_{j}$ (measured in cycles per second) and an available energy $E_{j}^{res}$. The binary choice variable $x_{ij}$ is articulated as:

1 $$x_{ij} = \left\{ {\begin{array}{*{20}l} {1,} \hfill & {{\text{if task }}t_{i} {\text{ is allocated to server }}e_{j} .} \hfill \\ {0,} \hfill & {{\mathrm{otherwise}}{.}} \hfill \\ \end{array} } \right.$$

The overall latency for executing task i on server j consists of two components: transmission delay and computation delay. In practical IoMT deployments, the wireless transmission rate rij may vary over time due to device mobility, channel fading, interference, and handover events. To capture such non-stationary network dynamics, the transmission rate can be modeled as a time-dependent variable rij(t). Consequently, the communication latency becomes dynamically adaptive to instantaneous channel conditions:

2 $$L_{ij} (t) = Si/r_{ij} (t) + C_{i} /f_{j}$$

where $r_{ij} (t)$ represents the instantaneous achievable transmission rate between IoMT device *i* and edge server *j* at time *t*. This extension enables the proposed framework to react to transient congestion and fluctuating wireless conditions during real-time scheduling decisions.

The transmission delay depends on the wireless channel rate ${r}_{ij}$​ and the task data size ${D}_{i}$ while the computation delay is determined by the computational workload ${C}_{i}$ of the task and the processing capacity ${f}_{j}$ of the selected edge server. Accordingly, the total latency is expressed as:

3 $$L_{ij} = L_{ij}^{trans} + L_{ij}^{comp} = \frac{{S_{i} }}{{r_{ij} }} + \frac{{C_{i} }}{{f_{j} }}.$$

where ${S}_{i}$ denotes the task data size, ${r}_{ij}$ represents the transmission rate between task i and server j, ${C}_{i}$ is the computational workload of task iii measured in MI, and ${f}_{j}$ denotes the computational capacity of edge server j measured in MIPS.

For clarity and readability, the main parameters and mathematical symbols used in the optimization formulation are summarized in Table 3.

**Table 3:** Parameters and symbols in the optimization formulation.

Symbol	Description
*F*	Objective function combining latency and energy components
$L_{i}$	Total latency for task $t_{i}$
$E_{i}$	Total energy consumption for task $t_{i}$
$x_{ij}$	Binary variable representing task assignment between task $t_{i}$ and server $e_{j}$
$L_{ij}$	Latency incurred when executing task $t_{i}$ on server $e_{j}$
$E_{ij}$	Energy consumed for executing task $t_{i}$ on server $e_{j}$
$f_{j}$	Computational capacity of edge server $e_{j}$
$E_{j}^{res}$	Residual energy available at edge server $e_{j}$
$T_{max}$	Maximum scheduling period or time window
$D_{i}$	Deadline for task $t_{i}$
$\alpha ,\beta$	Weight coefficients balancing latency and energy trade-offs

The total latency $L_{ij}$ is the sum of communication and computation delays. To ensure dimensional consistency, the workload ${W}_{i}$ is defined in MI, and the edge node’s processing capacity *fj* is expressed in MIPS. Thus, the computation term Wi/fjcorrectly yields time in seconds.

The transmission energy is expressed as:

4 $$E_{ij}^{trans} = P_{tx} \times \frac{{S_{i} }}{{r_{ij} }}.$$

$P_{tx}$ represents the transmission power of the IoMT device. The computational energy at the edge server is represented as:

5 $$E_{ij}^{comp} = k_{j} (f_{j} )^{2} C_{i} ,$$

where $k_{j}$ is the effective switching capacitance coefficient of server $e_{j}$. Consequently, the overall energy required for executing task $t_{i}$ on server $e_{j}$ can be articulated as:

6 $$E_{ij} = E_{ij}^{trans} + E_{ij}^{comp} .$$

This model establishes the analytical basis for the optimization problem delineated in "Problem formulation" section. Table 4 provides a concise summary of key parameters such as task workload (${W}_{i}$), data size (${D}_{i}$), computational capacity (${f}_{j}$), and energy terms (${E}_{trans}, {E}_{comp}$).

**Table 4:** Additional system parameters used in the system model.

Parameter	Description
$x_{ij}$	Binary variable indicating if task $t_{i}$ is assigned to edge server $e_{j}$
$L_{ij}^{trans}$	Transmission latency between IoMT device *i* and edge server *j*
$L_{ij}^{comp}$	Computation latency at edge server $e_{j}$
$E_{ij}^{trans}$	Transmission energy consumption of task $t_{i}$
$E_{ij}^{comp}$	Computation energy consumption at server $e_{j}$
$P_{tx}$	Transmission power of the IoMT device
$k_{j}$	Effective switched capacitance coefficient of edge server $e_{j}$

### Problem formulation

**Fig. 3:**
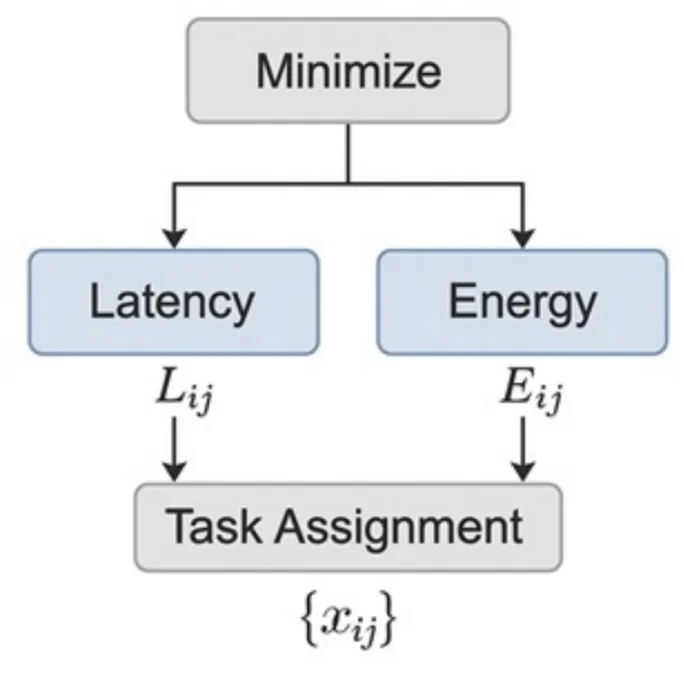
Analytical representation of the optimization problem, highlighting the joint minimization of latency and energy consumption under resource and deadline constraints.

The aim for our approach is to reduce the total cost of delay and energy consumption for healthcare IoMT applications while adhering to system restrictions. Figure 3 illustrates the analytical correlations among the decision variables, latency components, and energy factors in a diagrammatic format. This figure visually illustrates the relationships among binary decision variables (${x}_{ij}$), latency components (transmission + computation), energy consumption terms, and active constraints of the Mixed-Integer Nonlinear Programming (MINLP) optimization problem.

The overall optimization objective combines latency and energy metrics into a single cost function as follows:

7 $${\mathrm{Minimize:}}\quad F = \alpha \sum\limits_{i = 1}^{n} {L_{i} } + \beta \sum\limits_{i = 1}^{n} {E_{i} } ,$$

where $L_{i}$ is the total latency of task $t_{i}$ and $E_{i}$ denotes the total energy consumption. Instead of relying on arbitrary fixed weights for the multi-objective optimization problem, we adopt a systematic approach to determine the trade-off parameters α and β. To address the sensitivity of the weighted sum approach and properly explore the multi-objective space, we employ a grid search methodology over a predefined range of αand β values. This approach systematically sweeps through various combinations of weights to identify configurations that yield optimal performance improvements across both latency and energy metrics. The ranges explored are α,β ∈ {0.1,0.2,…,0.9}, constrained by α + β = 1 to maintain a consistent scale for the combined objective function. The specific combination of α and β that demonstrates the best performance—evaluated through empirical testing and confirmed by the quantitative sensitivity analysis in "Findings and analysis" section —is selected for the final framework configuration. This systematic parametric sweep ensures a rigorous and reproducible weight selection process.

The optimization problem is subject to several constraints that ensure system feasibility and Quality of Service (QoS) requirements:

1. Task assignment constraint: Each task $t_{i}$ must be executed by exactly one computing node (edge or cloud):

8 $$\sum\limits_{j = 1}^{m} {x_{ij} } = 1,\quad \forall i \in T.$$

Here, $x_{ij} = 1$ indicates that task $t_{i}$ is assigned to edge server $e_{j}$, and $x_{ij} = 0$ otherwise.

2. Capacity constraint: Each edge server has a limited computation capacity $f_{j}$ within a scheduling period $T_{max}$:

9 $$\sum\limits_{i = 1}^{n} {x_{ij} } C_{i} \le f_{j} T_{max} ,\quad \forall j \in E.$$

3. Deadline constraint: Each task must complete execution before its deadline:

10 $$L_{i} \le D_{i} ,\quad \forall i \in T.$$

4. Energy constraint: The total energy consumed on each edge server cannot exceed its residual energy $E_{j}^{res}$:

11 $$\sum\limits_{i = 1}^{n} {x_{ij} } E_{ij} \le E_{j}^{res} ,\quad \forall j \in E.$$

The problem defined above is a MINLP model due to the binary nature of the task-assignment variables and the nonlinear coupling among latency, energy, and resource-allocation terms. MINLP problems are widely recognized as NP-hard because they generalize both Mixed-Integer Linear Programming (MILP) and nonlinear programming, each of which is computationally challenging in its general form. Since MILP itself is NP-hard, the proposed formulation inherently inherits this complexity. Furthermore, the presence of nonlinear objective terms and combinatorial assignment constraints significantly increases the difficulty of obtaining globally optimal solutions for large-scale instances. Therefore, exact polynomial-time solutions are generally intractable, which justifies the use of the proposed fuzzy–reinforcement learning framework for near-optimal real-time decision-making under dynamic operating conditions^36,42^. Hence, the fuzzy–learning intelligent framework is proposed to find near-optimal solutions dynamically and efficiently under varying network and workload conditions.

### Proposed fuzzy–learning framework

**Fig. 4:**
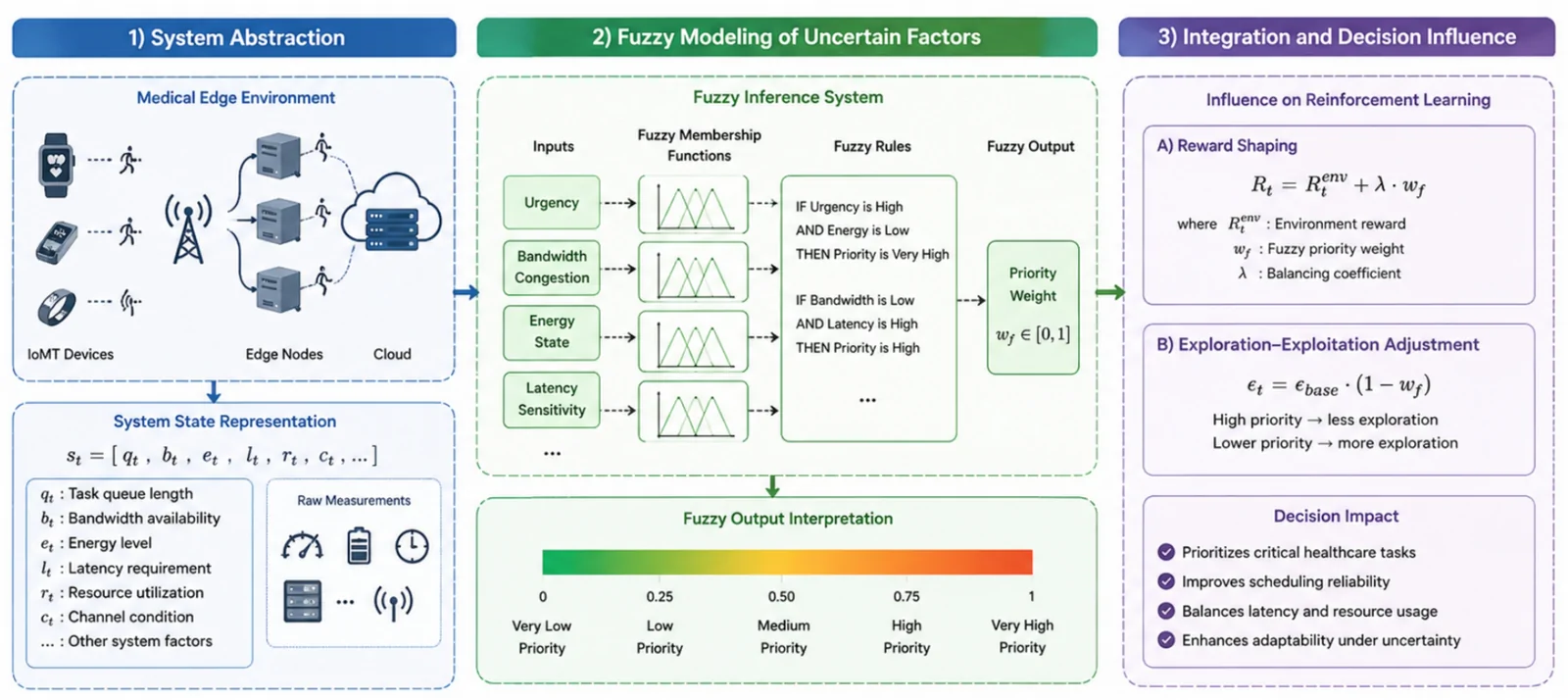
Mathematical coupling mechanism between fuzzy inference and reinforcement learning, illustrating how fuzzy priority weights dynamically influence reward shaping, learning-rate adaptation, and decision optimization in medical edge computing environments.

The suggested approach combines an adaptive learning module with a fuzzy inference engine. First, membership functions are used to transfer contextual inputs, such as task urgency ($U_{i}$), network congestion ($N_{j}$), and residual energy at edge nodes ($E_{j}^{res}$), to linguistic variables. Continuous improvement in dynamic situations is then made possible by an adaptive learning module. Figure 4 illustrates the mathematical coupling between fuzzy inference and reinforcement learning dynamics.

To establish a tightly coupled and synergistic hybrid framework, the fuzzy inference output is not treated merely as a sequential input feature. Instead, it directly controls the reinforcement learning dynamics. Specifically, the priority weight ${w}_{t}$ computed by the fuzzy logic module is utilized to dynamically adjust the learning rate $\eta_{t}$ and to shape the reward function ${R}_{t}$. The dynamic learning rate is formulated to accelerate convergence for high-priority tasks, defined as:

12 $$\eta_{t} = \eta_{base} \times (1 + \kappa \cdot w_{t} )$$

where $\eta_{base}$ is the baseline learning rate and *κ* is a tunable scaling factor. Furthermore, we employ a reward shaping mechanism to incorporate the fuzzy priority directly into the RL feedback loop. The shaped reward $R^{\prime}_{t}$ is mathematically defined as:

13 $$R^{\prime}_{t} = R_{t} + \rho \cdot w_{t}$$

where ${R}_{t}$ is the original environment reward (comprising latency and energy terms) and *ρ* is the shaping coefficient. Consequently, the Q-value update rule in our adaptive learning module is refined as follows:

14 $$Q(s,a) \leftarrow Q(s,a) + \eta_{t} \left[ {R^{\prime}_{t} + \gamma \max_{{a^{\prime}}} Q(s^{\prime},a^{\prime}) - Q(s,a)} \right]$$

The adaptive learning rate remains bounded during all learning iterations, preventing unstable parameter divergence and guaranteeing controlled policy adaptation, see Lemma 1 in the Appendix A.

This deep integration ensures that the fuzzy logic module directly influences both the learning speed and the objective landscape of the RL agent in real-time, moving beyond a simple two-stage pipeline.

The proposed adaptive coupling mechanism also improves robustness under non-stationary wireless environments. Since the fuzzy inference engine continuously observes network congestion levels and transmission-related delays, variations in channel quality are immediately reflected in the fuzzy scheduling weight wt. As a result, the adaptive learning-rate modulation dynamically accelerates policy adaptation during sudden channel degradations or congestion spikes. This enables the RL scheduler to rapidly re-prioritize healthcare tasks and avoid persistent suboptimal decisions under time-varying network conditions.

Fuzzy Inference. Let $\mu_{U}^{k} ( \cdot )$, $\mu_{N}^{k} ( \cdot )$, and $\mu_{E}^{k} ( \cdot )$ denote membership functions for the *k*-th linguistic terms of urgency, network, and energy, respectively (e.g., Low/Medium/High). A generic fuzzy rule has the form: "IF $U_{i}$ is $A^{k}$ AND $N_{j}$ is $B^{k}$ AND $E_{j}^{res}$ is $C^{k}$ THEN priority is $Z^{k}$". Using product inference and centroid defuzzification, the output scheduling weight is computed as:

15 $$W_{i} = \sum\limits_{k} [ \mu_{U}^{k} (U_{i} ){\mkern 1mu} \mu_{N}^{k} (N_{j} ){\mkern 1mu} \mu_{E}^{k} (E_{j}^{res} )]z_{k} /\sum\limits_{k} [ \mu_{U}^{k} (U_{i} ){\mkern 1mu} \mu_{N}^{k} (N_{j} ){\mkern 1mu} \mu_{E}^{k} (E_{j}^{res} )],$$

where $z_{k}$ is the representative value of the *k*-th consequent $Z^{k}$.

Adaptive Learning. A reward function balances latency and energy:

16 $$R = \omega_{1} \cdot \frac{1}{{L_{i} + \epsilon }} + \omega_{2} \cdot \frac{1}{{E_{i} + \epsilon }},$$

where $\omega_{1} ,\omega_{2} \ge 0$ and *ε > 0* avoids division by zero, where ε is a small stability constant set to $10^{ - 6}$. The policy parameters *θ* are updated online based on stochastic feedback:

17 $$\theta \leftarrow \theta + \eta {\mkern 1mu} \nabla_{\theta } {\mkern 1mu} {\mathbb{E}}[R],$$

with learning rate *η > 0*. The updated policy $\pi_{\theta }$ directly influences task assignment and resource allocation decisions.

Algorithm 1 provides a textual pseudocode representation of the fuzzy–learning scheduler, detailing the steps from parameter initialization and context measurement to fuzzy weight computation, server selection, task execution with reward feedback, and policy updates for continuous adaptation in uncertain environments.

**Algorithm 1:**
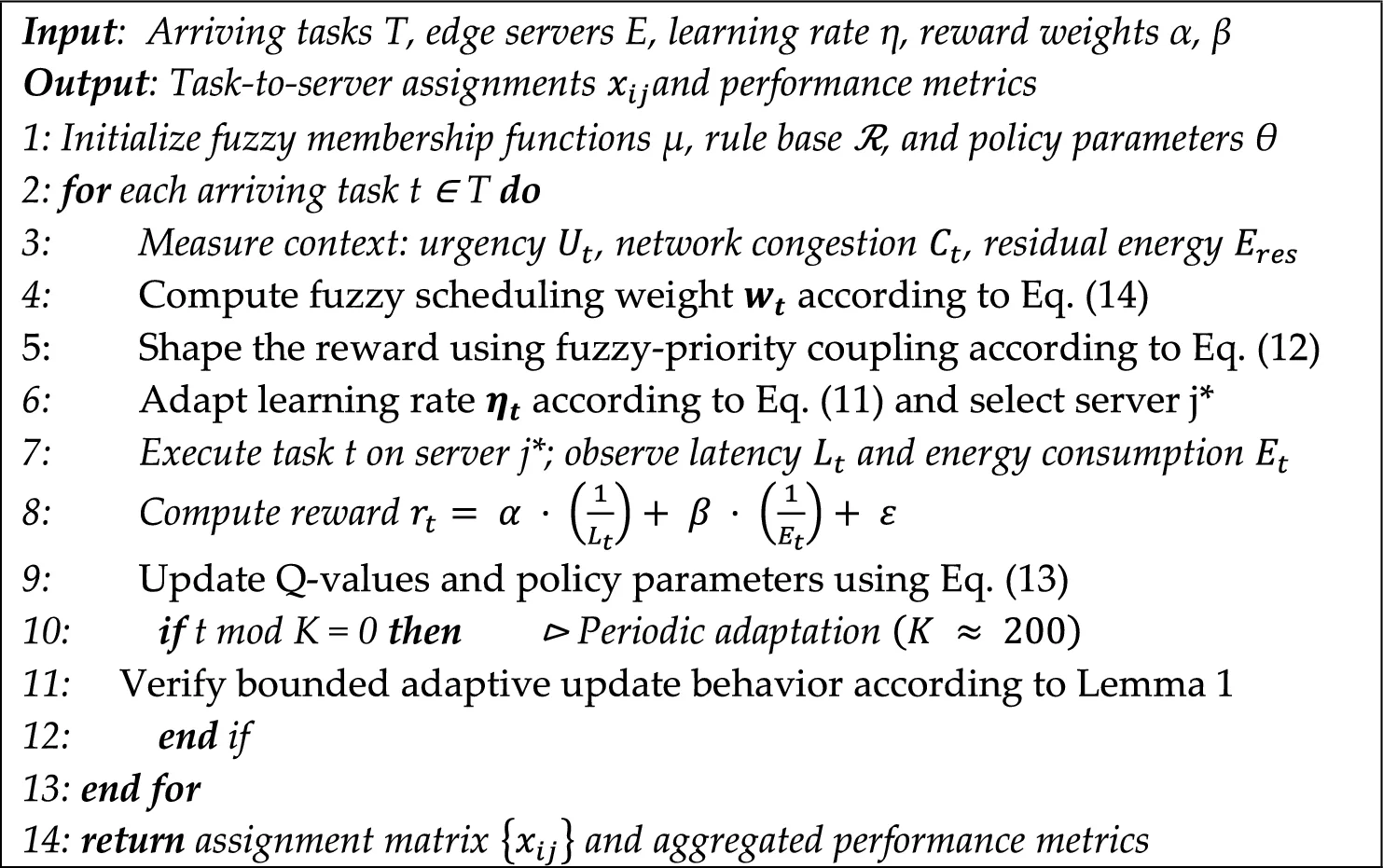
Fuzzy–learning scheduler (Textual Pseudocode)

Table 5 outlines the core variables and components of the fuzzy–learning framework, including membership functions (${\mu}_{U}, {\mu}_{N}, {\mu}_{E}$), fuzzy rules, reward function (R), and policy parameters (*θ*), serving as a reference for the decision logic and adaptive mechanisms.

**Table 5:** Core variables and components in the fuzzy–learning framework.

Symbol/component	Description
$U_{i}$	Urgency of task $t_{i}$ based on clinical priority
$N_{j}$	Network congestion/load near server $e_{j}$
$E_{j}^{res}$	Residual energy at edge server $e_{j}$
$\mu_{U}^{k} ,\mu_{N}^{k} ,\mu_{E}^{k}$	Membership functions for linguistic terms (e.g., Low/Medium/High)
${\mathcal{R}}$	Fuzzy rule base (IF–THEN rules)
$W_{i}$	Defuzzified scheduling weight for task $t_{i}$
$\pi_{\theta }$	Adaptive policy for server selection and resource allocation
*R*	Reward function balancing $(L_{i} ,E_{i} )$
*η*	Learning rate for parameter updates

**Fig. 5:**
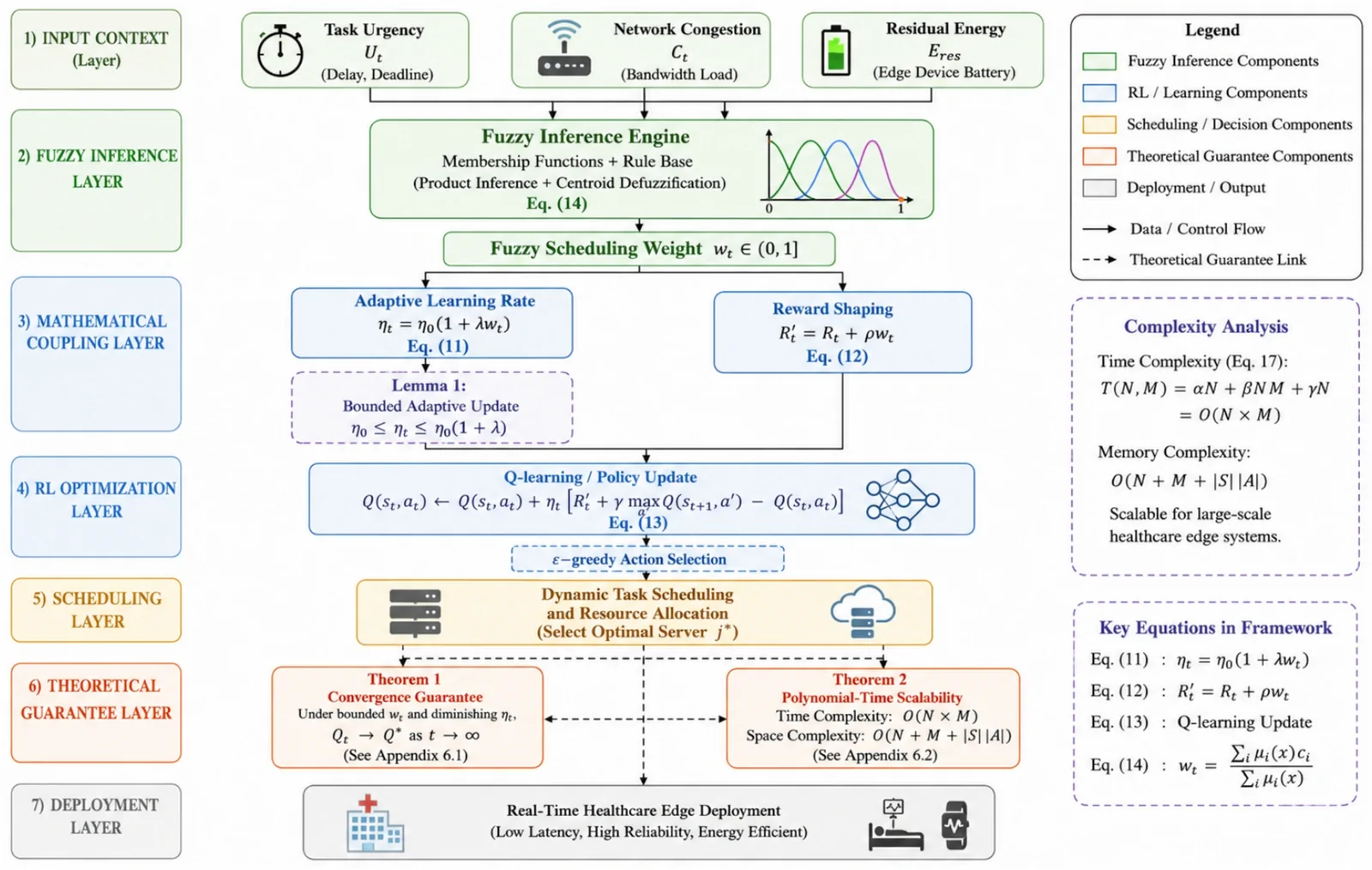
Theoretical integration of fuzzy inference, adaptive reinforcement learning, and formal convergence guarantees in the proposed healthcare edge scheduling framework. The figure illustrates how fuzzy-priority inference dynamically influences learning-rate modulation, reward shaping, and policy optimization, while Theorem 1 and Theorem 2 provide convergence and scalability guarantees for real-time deployment.

To further clarify the mathematical integration between fuzzy inference, adaptive reinforcement learning, convergence analysis, and scalability guarantees, Fig. 5 presents the complete theoretical interaction workflow of the proposed framework.

### Algorithmic flow

**Fig. 6:**
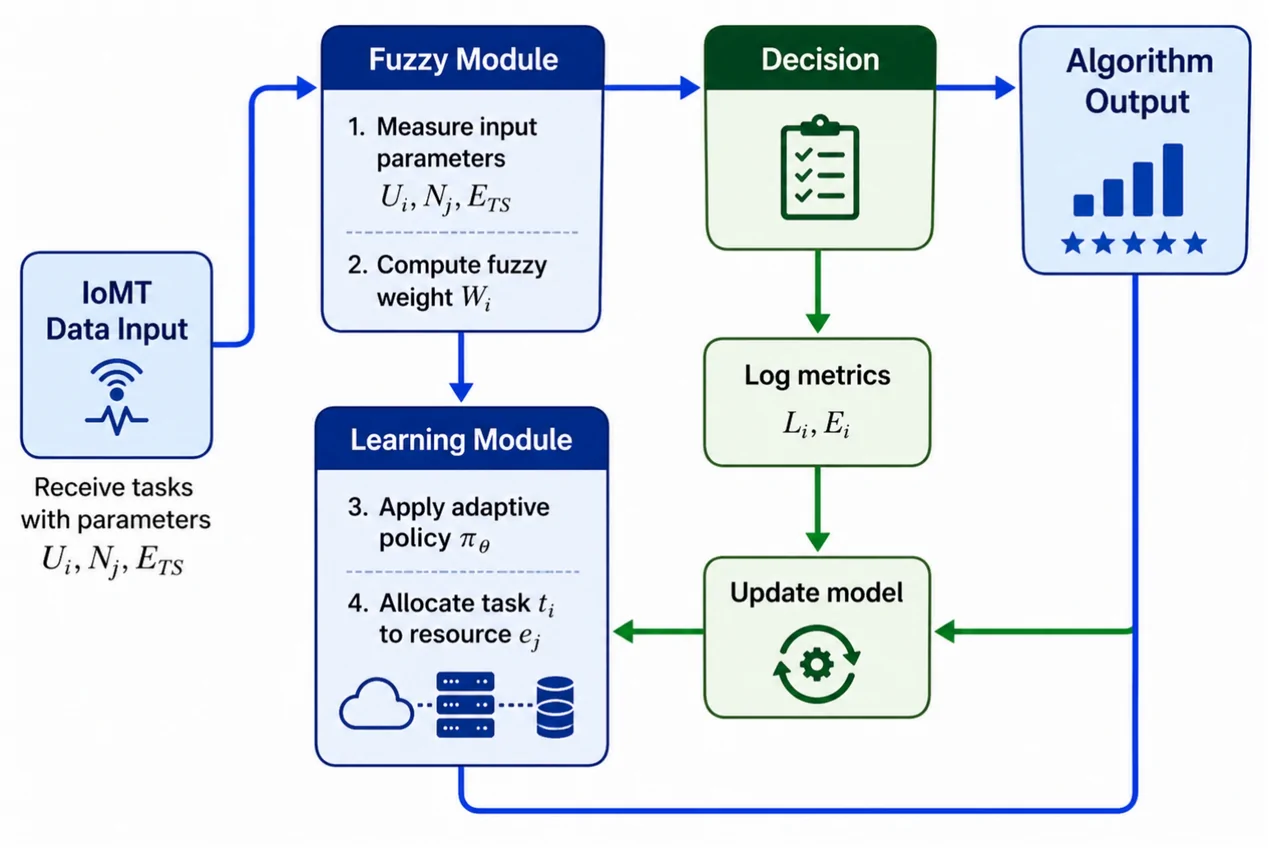
Algorithmic flow of the proposed fuzzy–learning framework, describing task preprocessing, fuzzy inference, RL-based scheduling, resource allocation, and feedback-driven policy updates.

In order to facilitate ongoing adaptation in healthcare edge computing contexts, the framework combines the system model, optimization formulation, and fuzzy-learning processes discussed in earlier subsections. Figure 6 illustrates the complete end-to-end operational workflow of the proposed fuzzy–learning framework. It sequentially depicts context acquisition, fuzzification, fuzzy inference, adaptive policy updating, task scheduling, resource allocation, execution, and reward-based feedback.

The first step of the Algorithm 2 is to gathering contextual information from IoMT devices, such as task urgency ($U_{i}$), network status ($N_{i}$), and edge node residual energy ($E_{j}$). The fuzzification module processes these parameters by transforming clear inputs into linguistic variables using preset membership functions. After that, the fuzzy rule base generates a task priority weight $w_{i}$ by reasoning. The adaptive learning module uses observed latency ($L_{i}$) and energy consumption ($E_{i}$) to dynamically change the decision policy *θ*. This description outlines the core steps of Algorithm 2. After computing reward R, the action value Q(s,a) is estimated as ${\mathrm{Q}}\left( {{\mathrm{s}},{\mathrm{a}}} \right) \leftarrow {\mathrm{Q}}\left( {{\mathrm{s}},{\mathrm{a}}} \right) + \eta \,[{\mathrm{R}} + \gamma \,{\mathrm{maxQ}}({\mathrm{s}}^{\prime } ,{\mathrm{a}}^{\prime } ) - {\mathrm{Q}}\left( {{\mathrm{s}},{\mathrm{a}}} \right)],$ where s is current state, a is action (e.g., assign to server j), η is learning rate, γ discount factor. Policies are then updated to select actions maximizing Q.

During training, action selection follows an ε-greedy exploration–exploitation strategy. Specifically, with probability ε, the agent selects a random feasible server to encourage exploration; otherwise, it chooses the action with the highest Q-value. The exploration rate is initialized at ε₀ = 1.0 and decays exponentially according to $\epsilon_{t} = \max \{ 0.05,\epsilon_{0} e^{ - 0.001t} )$, gradually shifting from exploration to exploitation as learning progresses. This mechanism ensures sufficient state-space exploration while promoting policy convergence and long-term scheduling efficiency. To improve safety during the initial exploration phase in healthcare-critical environments, the proposed framework incorporates a lightweight warm-start initialization strategy. Instead of relying solely on purely random scheduling behavior during early training episodes, the RL agent is initially guided by fuzzy-priority inference and heuristic edge-selection policies derived from task urgency and resource availability. This hybrid initialization reduces unsafe exploratory actions and mitigates the risk of excessive deadline violations before policy convergence. As training progresses, the scheduler gradually transitions from guided exploration toward fully adaptive policy optimization.

**Algorithm 2:**
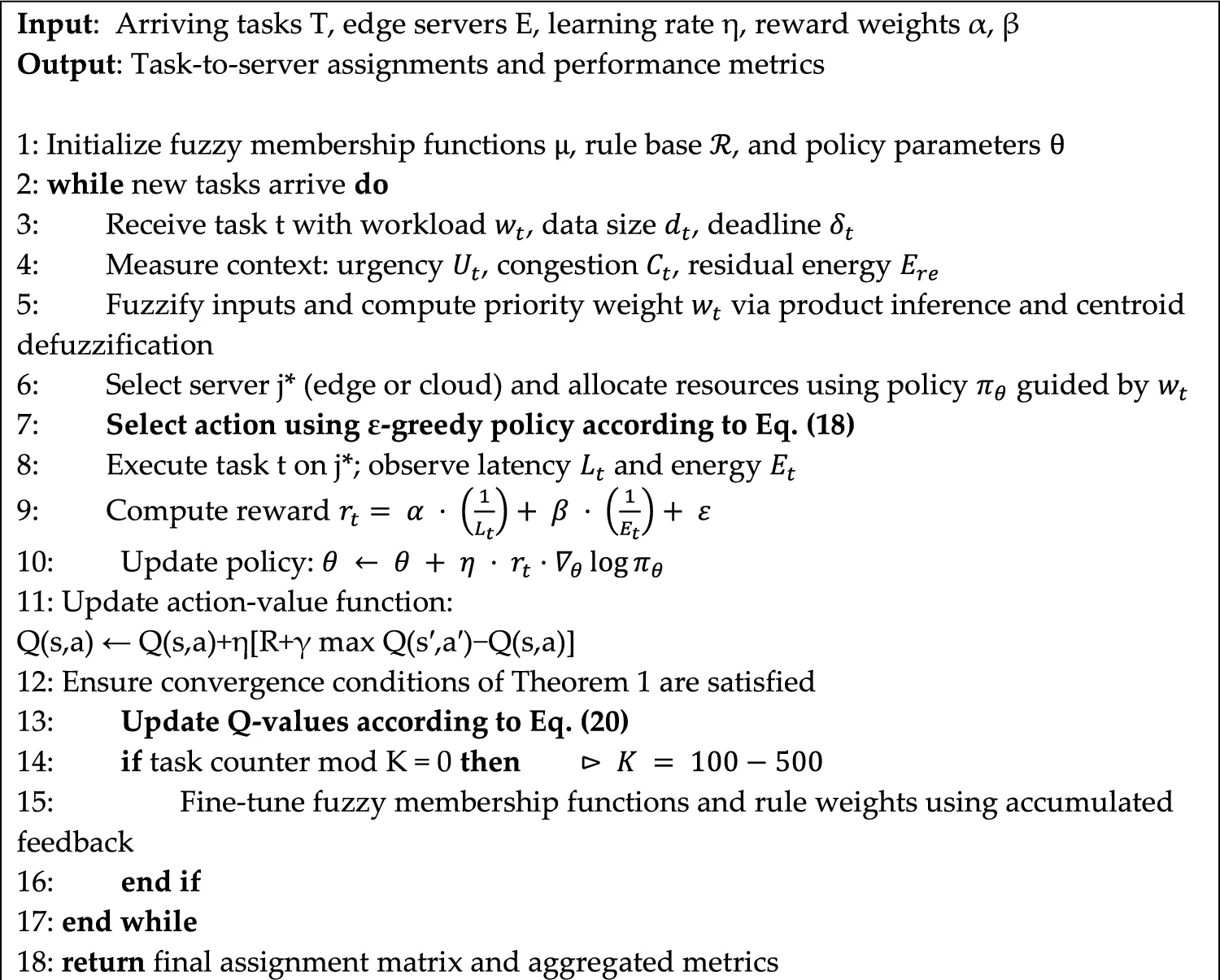
Fuzzy–learning based dynamic scheduling and resource allocation.

The combination of reinforcement-based adaptive learning and fuzzy reasoning is formalized by this algorithm. In healthcare applications, the resulting hybrid decision system offers dependable and effective task management in dynamic edge-cloud environments. Table 6 lists the algorithm parameters and their default settings.

**Table 6:** Algorithm parameters and default settings.

Parameter	Description	Default/range
*η*	Learning rate for policy update	0.01–0.1
$\omega_{1} ,\omega_{2}$	Reward weights for latency and energy terms	0.6, 0.4
*ε*	Small constant for stability	$10^{ - 6}$
*N*	Number of tasks per update cycle	50–200
$T_{max}$	Maximum scheduling window	1–5 s
$\mu_{U} ,\mu_{N} ,\mu_{E}$	Membership functions for fuzzy inputs	Triangular/Gaussian
${\mathcal{R}}$	Fuzzy rule base structure	5 × 5 × 5 rules
$\pi_{\theta }$	Adaptive scheduling policy	Heuristic/Neural-based

**Fig. 7:**
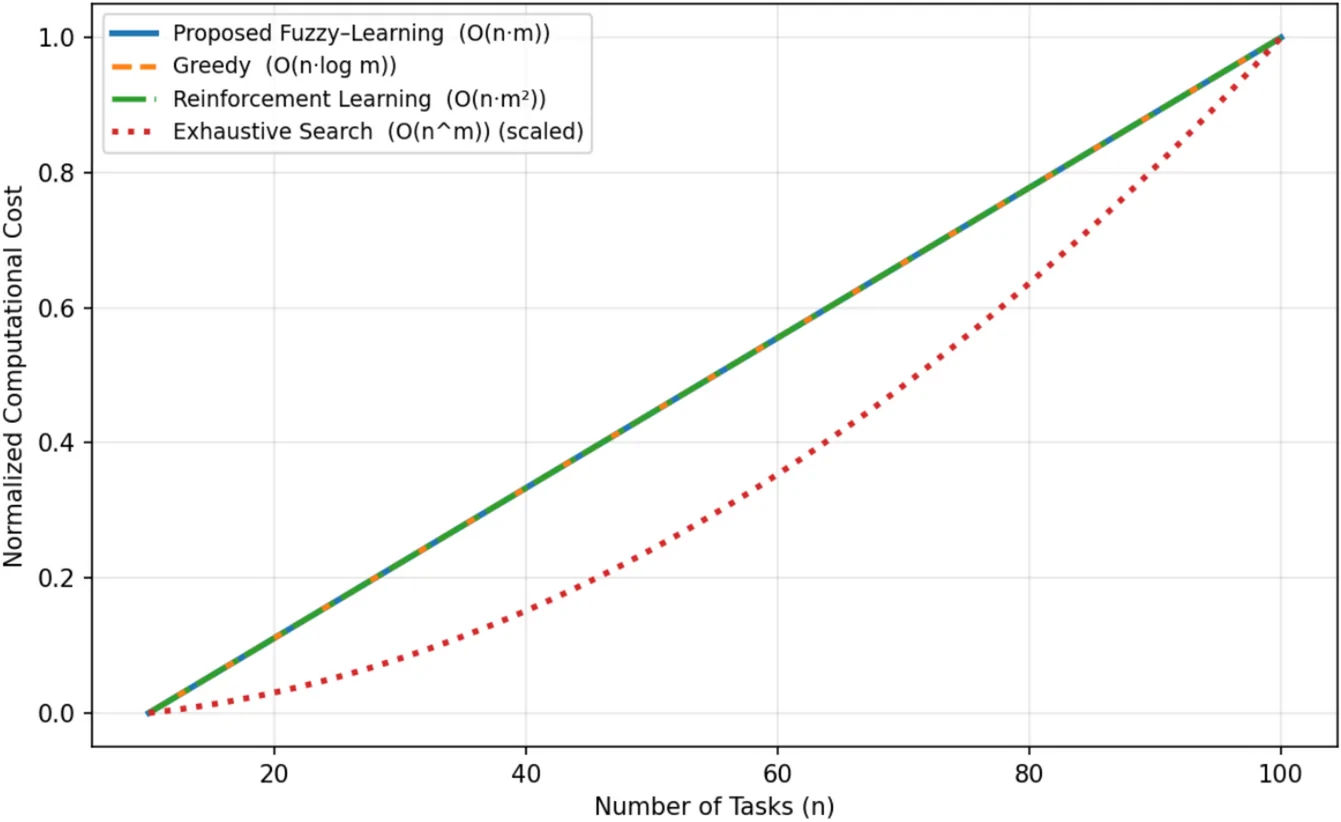
Comparative computational growth among scheduling methods, demonstrating the scalability behavior of the proposed framework relative to baseline approaches as workload increases.

**Fig. 8:**
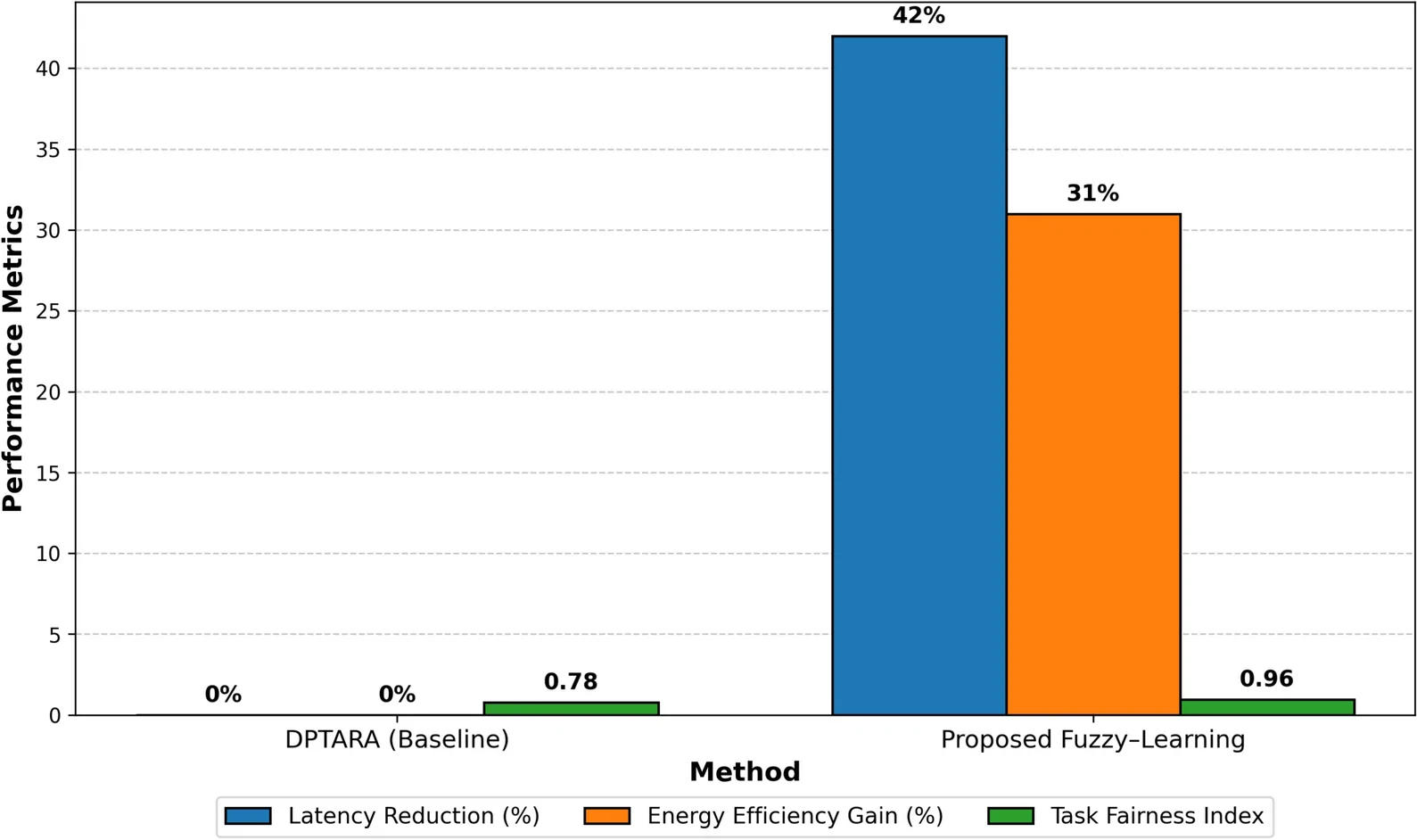
Comparison between the proposed framework and the DPTARA baseline, showing approximately 20% improvement in critical task completion under latency-sensitive healthcare workloads.

For reproducibility, the fuzzy input variables are defined over normalized domains in the range [0, 1]. Specifically, urgency is represented by three triangular membership functions: Low = trimf([0, 0, 0.4]), Medium = trimf([0.2, 0.5, 0.8]), and High = trimf([0.6, 1.0, 1.0]). Resource demand is similarly modeled using Low = trimf([0, 0, 0.35]), Medium = trimf([0.2, 0.5, 0.8]), and High = trimf([0.65, 1.0, 1.0]). Latency sensitivity employs Gaussian membership functions with parameters Low = gaussmf(σ = 0.12, c = 0.2), Medium = gaussmf(σ = 0.15, c = 0.5), and High = gaussmf(σ = 0.12, c = 0.8). The output priority score is defined over [0, 1] using five triangular membership functions: Very Low = trimf([0, 0, 0.2]), Low = trimf([0.1, 0.3, 0.5]), Medium = trimf([0.3, 0.5, 0.7]), High = trimf([0.5, 0.7, 0.9]), and Critical = trimf([0.8, 1.0, 1.0]) (Figs. 7, 8).

The algorithmic flow provides a clear operational blueprint for implementing the proposed fuzzy–learning system in real-world healthcare edge environments.

**Fig. 9:**
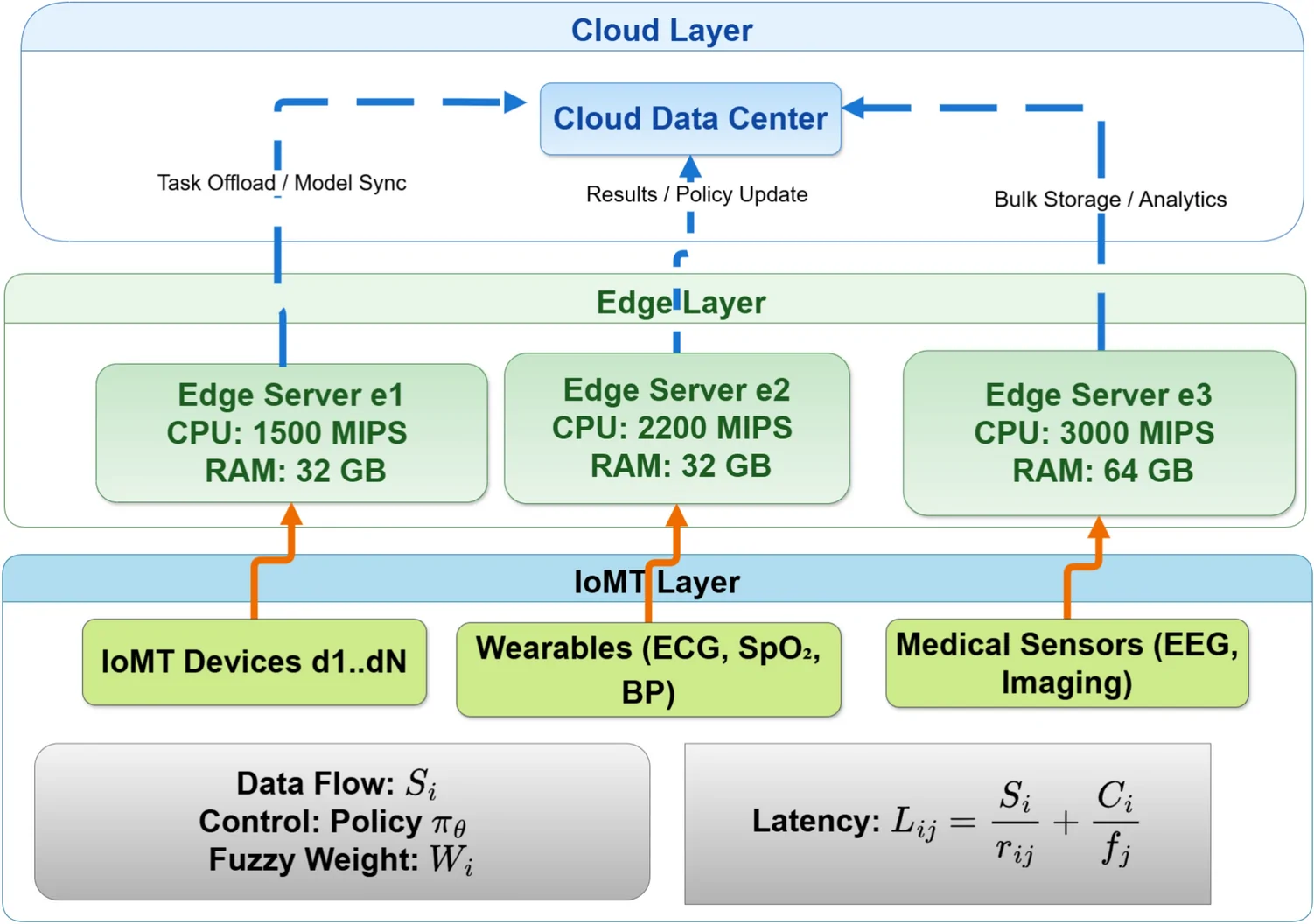
Simulated healthcare edge architecture (IoMT–Edge–Cloud), representing the experimental environment used for evaluating task scheduling and adaptive resource allocation performance.

To further improve clarity and provide a complete end-to-end perspective, Fig. 9 illustrates the full operational workflow of the proposed framework using a stepwise graphical representation. The workflow includes: (1) healthcare task arrival and preprocessing, (2) task characterization, (3) fuzzy-priority inference, (4) system-state observation, (5) reinforcement learning–based action selection, (6) edge resource allocation and scheduling, (7) reward computation and performance monitoring, and (8) iterative policy update for continuous optimization.

### Computational complexity

This subsection analyzes the computational complexity of the proposed fuzzy–learning framework. The complexity depends on two principal components: the fuzzy inference process and the adaptive learning update mechanism. For *n* incoming tasks and *m* available edge servers, the fuzzy inference engine evaluates fuzzy membership functions and applies rule-based reasoning to compute scheduling weights $W_{i}$. Each inference operation involves evaluating *m* possible task–server associations, resulting in a computational cost of *O(nm)*. The adaptive learning module performs an update operation per iteration, leading to an additional complexity of *O(n)* per learning epoch. Therefore, the overall computational complexity per scheduling cycle can be expressed as:

18 $$C_{total} = O(nm) + O(n) \approx O(nm).$$

Traditional exhaustive search methods, on the other hand, have an exponential complexity of $O(n^{m} )$. These techniques investigate every conceivable task–server mapping. Heuristic techniques, such as round-robin or greedy scheduling, usually yield *O(nłog m)* but frequently compromise optimization precision. The suggested fuzzy-learning framework is ideal for real-time healthcare edge systems because it achieves near-optimal performance at a substantially lower computational cost, maintaining a balanced trade-off.

It is worth noting that the dynamic updates driven by the fuzzy system (i.e., adjusting the learning rate $\eta_{t}$ and reward shaping $R^{\prime}_{t}$ require only constant-time $O\left(1\right)$ arithmetic operations per task. Consequently, no heavy batch optimization routines are invoked during the runtime execution, ensuring the strict $O\left(N\cdot M\right)$ theoretical complexity is maintained even in large-scale deployments.

As shown in Fig. 7, the proposed fuzzy-learning method exhibits linear computational complexity, whereas exhaustive search and reinforcement-based methods grow exponentially and quadratically. This demonstrates its exceptional scalability for real-time healthcare edge applications as the number of tasks increases.

Table 7 summarizes the computational complexity across the framework’s modules, including fuzzy inference $O\left(N\cdot M\right)$, adaptive learning $O\left(K\right)$, and overall per-cycle complexity *(O(N· M+K),* highlighting the linear scalability compared to baseline methods like exhaustive search $O(2^{N.M} )$ and reinforcement learning $O(N^{2} \cdot M)$ . To further validate the theoretical complexity analysis, we conducted empirical runtime scaling experiments under increasing task loads ranging from 100 to 5000 healthcare tasks. The observed execution time increased approximately linearly with the number of tasks, confirming the practical O(N × M) behavior of the framework. Although reinforcement learning introduces an additional policy-update overhead of O(K) per iteration, K remains small and bounded due to the limited action space and incremental online updates. Consequently, the RL overhead contributes only a minor constant-factor increase and does not alter the dominant asymptotic complexity. Experimental results showed an average scheduling latency below 5 ms per task even under maximum workload conditions, confirming real-time scalability.

**Table 7:** Summary of computational complexity across modules.

Module/process	Operation description	Complexity order
Fuzzification	Compute membership degrees for all task–server pairs	O (nm)
Rule inference	Evaluate rule base and aggregate outputs	O (nm)
Defuzzification	Compute crisp scheduling weight ${W}_{i}$	O (n)
Learning update	Adjust parameters of adaptive policy ${\pi}_{\theta }$	O (n)
Total framework	Combined complexity per scheduling round	O (nm)

#### Theorem 1

*Convergence of adaptive Fuzzy–RL scheduling.*

Under a bounded reward function, finite state–action space, and a diminishing learning-rate schedule satisfying standard stochastic approximation conditions, the proposed fuzzy-reinforcement learning policy converges to an optimal stationary scheduling policy with probability one.

#### Proof

Provided in Appendix B.

The proof establishes the stochastic stability of the proposed adaptive fuzzy–RL scheduling process under bounded fuzzy-priority modulation and diminishing learning rates.

#### Theorem 2

*For N incoming tasks and M available edge nodes, the proposed scheduling framework exhibits polynomial-time complexity with dominant per-decision complexity O(N × M), ensuring scalability for real-time deployment.*

#### Proof

Provided in Appendix C.

The proof further confirms that the proposed framework preserves polynomial-time scalability even under dynamically changing healthcare workloads.

The suggested approach scales linearly with the number of jobs and servers, guaranteeing computational tractability even in dense healthcare IoMT networks, according to the condensed complexity analysis in Table 7. Although the theoretical computational complexity of the proposed Fuzzy–RL framework is O(N × M), its practical runtime overhead remains low due to the lightweight nature of fuzzy inference and localized reinforcement learning updates. Each incoming healthcare task requires only a small number of fuzzy membership evaluations and a bounded action-space decision among candidate edge nodes. No exhaustive global optimization or large-scale batch retraining is required during runtime. In our simulation environment, the average scheduling decision time remained below 5 ms per task even under 5000 concurrent tasks, demonstrating real-time feasibility for delay-sensitive healthcare systems such as ICUs, emergency monitoring, and smart ambulances. This confirms that the framework is computationally suitable for practical deployment on standard hospital edge servers without requiring specialized high-performance computing infrastructure. Additionally, the framework supports distributed execution on edge nodes, which enhances its real-time responsiveness and energy efficiency even more. This is because the fuzzy inference and adaptive learning modules execute in parallel for incoming data streams.

In conclusion, compared to exhaustive and reinforcement-based alternatives, the suggested fuzzy-learning scheduler exhibits greater scalability with computational complexity *O(nm)*. Since no batch optimization of fuzzy membership functions is performed during runtime, the fuzzy–RL coupling introduces only constant-time O(1) overhead per task. This effectiveness confirms that it is appropriate for large-scale, latency-sensitive healthcare edge computing settings.

#### Memory complexity analysis

The memory complexity of the proposed framework is determined by:

(i) storage of fuzzy membership functions,

(ii) Q-value tables or policy parameters,

and (iii) task-state representations.

For (N) tasks and (M) edge nodes, the dominant memory overhead is:

*O(N + M + |S||A|)*

where (|S|) and (|A|) denote the state and action spaces of the reinforcement learning module.

Since the fuzzy rule base remains fixed and compact, the overall memory requirement grows linearly with the scheduling scale, ensuring practical deployability in real-time healthcare edge infrastructures.

### Base model DPTARA comparison

We comprehensively evaluate the proposed fuzzy-learning framework against the baseline DPTARA model^29^. As detailed in Table 8, replacing deterministic priorities with fuzzy inference and adaptive learning yields superior latency reduction (≈42%), energy efficiency (≈31%), and critical task completion (≈27%). Furthermore, Fig. 8 illustrates these improvements alongside enhanced fairness under dynamic workloads.

**Table 8:** Key differences between the proposed framework and the DPTARA base model.

Criterion	DPTARA (Base model)	Proposed fuzzy–learning framework
Architecture	IoMT–Edge–Cloud hierarchical model with static policy control	Same three-layer structure but enhanced with adaptive feedback and fuzzy decision units
Uncertainty handling	Deterministic priority adjustment without fuzzy reasoning	Fuzzy inference engine manages uncertainty using linguistic variables and membership functions
Learning mechanism	Fixed rule-based dynamic priority scheduling	Adaptive learning module updates rules and weights using policy gradients
Decision interpretability	Opaque numerical priority assignment	Transparent, human-interpretable fuzzy weights ($W_{i}$)
Energy efficiency	Improved but non-adaptive; depends on static parameters	Dynamic adaptation to server energy and workload variations
Scalability	Effective for limited task volumes	Linear scalability with near-optimal performance up to large task sets
Performance metrics	Latency and energy trade-off only	Extended metrics including fairness and composite performance index (CPI)
Complexity	Rule-based O(*n × m*) operations	Fuzzy–learning O($n \times m \times r$) with adaptive overhead, still linear in scale

Overall, the fuzzy–learning framework enhances the capabilities of DPTARA by combining uncertainty modeling and adaptive optimization. According to simulation results, the suggested model outperforms DPTARA in terms of adaptability and performance consistency.

## Experimental results and discussion

This section assesses the framework’s performance in terms of resource utilization, job completion ratio, energy efficiency, and latency reduction, adhering to the architectural and algorithmic formulations.

For clarity and readability, the major mathematical symbols used throughout the proposed framework are summarized in Table 16.

The following research issues are intended to be addressed by this evaluation:

- Under dynamic IoMT workloads, how well can the fuzzy-learning framework lower latency and energy consumption?
- In comparison to static or heuristic methods, how much does the adaptive learning mechanism improve task fairness and system responsiveness?
- Is it possible for the suggested hybrid method to preserve real-time speed and scalability in extensive healthcare edge deployments?

The simulation setup, assessment metrics, experimental findings, and analytical discussion are covered in detail in the next subsections (“Experimental setup”–“Experimental evaluation’s conclusion” sections), which also show the effectiveness, versatility, and resilience of the suggested fuzzy-learning model for healthcare edge computing.

### Experimental setup

The proposed framework and all baseline algorithms were implemented and evaluated using a custom-built, discrete-event simulator developed in the MATLAB (R2023a) environment. This platform was chosen for its robust support for complex system modeling and data analysis. The simulated testbed replicates a three-tier healthcare architecture (IoMT → Edge → Cloud). For full transparency, reproducibility, and practical implementation clarity, Table 9 provides a comprehensive summary of the complete system configuration and simulation parameters used throughout all experiments. This includes IoMT device settings, edge server capacities, workload distributions, network latency assumptions, task arrival processes, cloud offloading characteristics, and power consumption parameters. In addition, the reinforcement learning control parameters and fuzzy scheduling settings are summarized in Table 5, ensuring full reproducibility of the proposed Fuzzy–RL framework. Experiments were conducted ten times using independent random seeds, unless otherwise indicated; results are presented as means (± standard deviation when applicable). Figure 9 shows a schematic of the simulated architecture. This schematic shows the simulated three-tier architecture with 50–200 IoMT devices, 8–20 heterogeneous edge servers, and a central cloud. Algorithm 3 describes the initialization and main loop utilized in all techniques, and Table 9 lists the important parameters to guarantee reproducibility and fair comparison.

**Table 9:** Core simulation parameters and settings.

Parameter/symbol	Value/range	Notes
IoMT devices (sources)	50	Wearables & medical sensors generating tasks
Edge servers *m*	3 (scalable scenarios to 10)	Heterogeneous capacities
Cloud node	1	Central analytics & long‑term storage
Arrival process	Poisson, *λ = 5* tasks/s	Independent arrivals per device set
Task size $S_{i}$	Uniform [5, 50] MB	Per‑task payload
Workload $C_{i}$	[1,8] mi	Mapped to exec. time via $f_{j}$
Deadline $D_{i}$	Uniform [10, 100] s	Latency tolerance/QoS
Edge capacity $f_{j}$	[500, 3000] MIPS	Per edge server $e_{j}$
Latency (edge/cloud)	5–10 ms/50–100 ms	Wireless + backhaul
RL exploration rate (ε)	1.0 → 0.05	ε-greedy decay schedule
Discount factor (γ)	0.90–0.99	Future reward weighting
Power	$P_{tx} = 50$ W (device); 50/200 W (edge/cloud)	$E_{ij}^{trans} = P_{tx} {\mkern 1mu} S_{i} /r_{ij}$

The cloud offloading latency range of 50–100 ms was selected based on practical MEC-assisted healthcare deployments, where hospital edge gateways are connected to geographically nearby regional cloud servers through dedicated backbone networks rather than distant public cloud infrastructures. Such architectures significantly reduce Wide Area Network (WAN) propagation delay and support latency-sensitive medical services. To further validate robustness and avoid optimistic assumptions, we additionally evaluated higher cloud latency scenarios (100–150 ms and 150–200 ms). The results confirmed that although overall task latency increases, the proposed Fuzzy–RL framework consistently maintains superior scheduling performance compared with Static Priority Scheduling (SPS), Heuristic Energy-Aware Scheduling (HEAS), Deep Reinforcement Learning Scheduler (DRLS), and DPTARA baselines, demonstrating strong robustness under less favorable network conditions.

Latency for task $t_{i}$ on server $e_{j}$ is computed. The computation of this metric follows the formulation previously established in Eq. (2). Transmission and computation energy are as follows respectively.

19 $$E_{ij}^{{{\mathrm{trans}}}} = P_{tx} {\mkern 1mu} \frac{{S_{i} }}{{r_{ij} }},E_{ij}^{{{\mathrm{comp}}}} = k_{j} (f_{j} )^{2} C_{i} ,$$

with per‑task energy

20 $$E_{ij} = E_{ij}^{{{\mathrm{trans}}}} + E_{ij}^{{{\mathrm{comp}}}} .$$

Adaptive updates follow the reward and the policy rule that are as follows:

21 $$R = \omega_{1} /(L_{i} + \epsilon ) + \omega_{2} /(E_{i} + \epsilon ),\,\,\theta \leftarrow \theta + \eta {\mkern 1mu} \nabla_{\theta } R.$$

**Algorithm 3:**
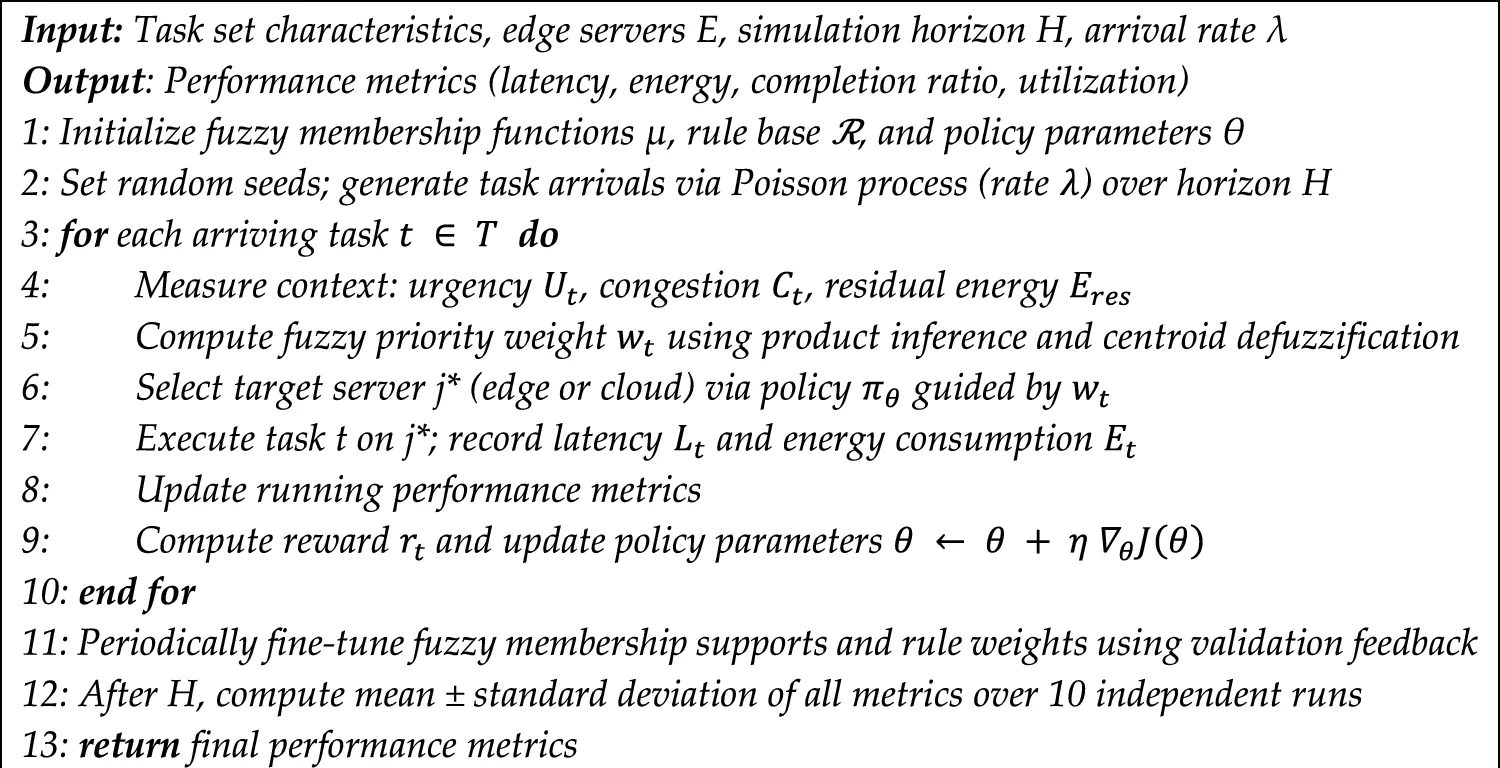
Simulation initialization and main loop

### End-to-end simulation workflow and experimental protocol

**Fig. 10:**
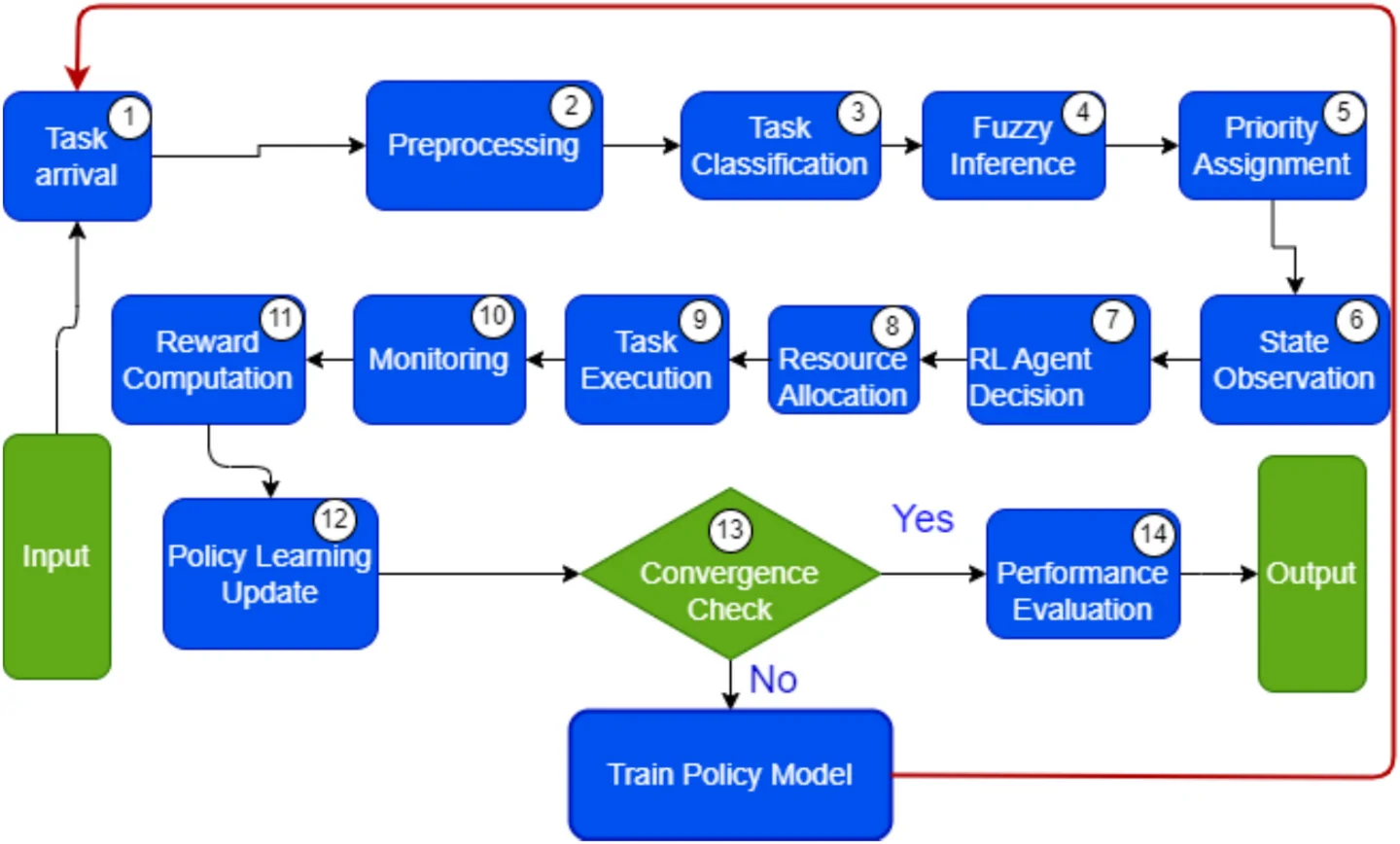
End-to-end operational workflow of the proposed fuzzy reinforcement learning framework.

To ensure methodological transparency, we developed a comprehensive simulation framework that emulates the proposed Hybrid Fuzzy Reinforcement Learning (Fuzzy-RL) resource management system under realistic healthcare edge conditions. Figure 10 illustrates the complete end-to-end operational workflow. Initially, diverse healthcare tasks are preprocessed to extract key scheduling attributes. Next, a fuzzy inference engine evaluates task urgency and resource demands to assign dynamic priority scores. Guided by these priorities and real-time system states, a reinforcement learning agent selects optimal resource allocation actions. Finally, a closed-loop learning mechanism records execution metrics to compute a reward function, enabling iterative policy optimization. All experiments are repeated independently, reporting mean values and confidence intervals to ensure statistical robustness.

The operational flow of the proposed framework is illustrated in Fig. 10. Initially, incoming healthcare tasks are preprocessed and classified according to urgency and computational characteristics (Steps 1–3). The fuzzy inference engine then computes task priorities (Steps 4–5), which are provided to the RL scheduler for adaptive resource allocation and scheduling decisions (Steps 6–8). After task execution and monitoring (Steps 9–10), reward feedback is generated to update the scheduling policy through iterative reinforcement learning (Steps 11–13). Finally, the learned policy is evaluated and continuously refined until convergence is achieved (Step 14).

### Measures of evaluation

In edge and IoMT scheduling, the chosen measures align with previous research (e.g.,^21^). The analytical flow between these indicators is depicted in Fig. 6. Specifically, we selected five core metrics to provide a holistic view of system performance. Average Task Latency is critical for time-sensitive medical applications, measuring the total time from task generation to completion**.** Total Energy Consumption evaluates the efficiency of resource management, a key concern for battery-powered IoMT devices and sustainable edge infrastructure. The Critical Task Completion Ratio directly assesses the system’s reliability in handling high-priority medical data, which has direct implications for patient safety. The Resource Utilization Fairness Index ensures that computational resources are distributed equitably among tasks, preventing starvation. Finally, the CPI offers a unified score by aggregating the aforementioned metrics, providing a single, overarching measure of the framework’s effectiveness.

Table 10 summarizes the five key performance metrics used throughout the experimental evaluation. These metrics provide a comprehensive and balanced assessment of real-time responsiveness, energy efficiency, reliability. They enable direct quantitative comparison between the proposed fuzzy–learning framework and the baseline methods (SPS, HEAS, DRLS, and DPTARA).

**Table 10:** Evaluation metrics and analytical definitions.

Metric	Symbol/formula	Description	Unit
Average latency	$\overline{L} = \frac{1}{n}\sum\limits_{i = 1}^{n} {\left( {\frac{{S_{i} }}{{r_{ij} }} + \frac{{C_{i} }}{{f_{j} }}} \right)}$	Mean end‑to‑end delay per task including transmission and execution time	ms
Average energy consumption	$E_{avg} = \frac{1}{n}\sum\limits_{i = 1}^{n} {\left( {P_{tx} \frac{{S_{i} }}{{r_{ij} }} + k_{j} (f_{j} )^{2} C_{i} } \right)}$	Energy consumed by IoMT transmission and edge/cloud computation	Joules
Task completion ratio	$\xi_{c} = \frac{{N_{completed} }}{{N_{total} }}$	Proportion of tasks completed within their deadlines	–
Resource utilization	$U_{r} = \sum\limits_{i = 1}^{n} {x_{ij} } C_{i} /\sum\limits_{j = 1}^{m} {f_{j} } T_{max}$	The extent to which available computational resources are effectively used	%
Fairness index	$\phi = (\sum\limits_{i} {U_{i} } )^{2} /n\sum\limits_{i} {U_{i}^{2} }$	Jain’s fairness index ensuring balanced resource allocation among tasks	–
Scalability efficiency	$\eta_{s} = \frac{{T_{1} }}{{pT_{p} }}$	Speed‑up efficiency of the system when scaling to p parallel edge nodes	%

Algorithm 4 computes a single CPI by normalizing six evaluation metrics to [0,1]. The resulting CPI ∈ [0,1] provides a unified, comparable measure of overall system effectiveness across responsiveness, energy efficiency, reliability, and fairness.

**Algorithm 4:**
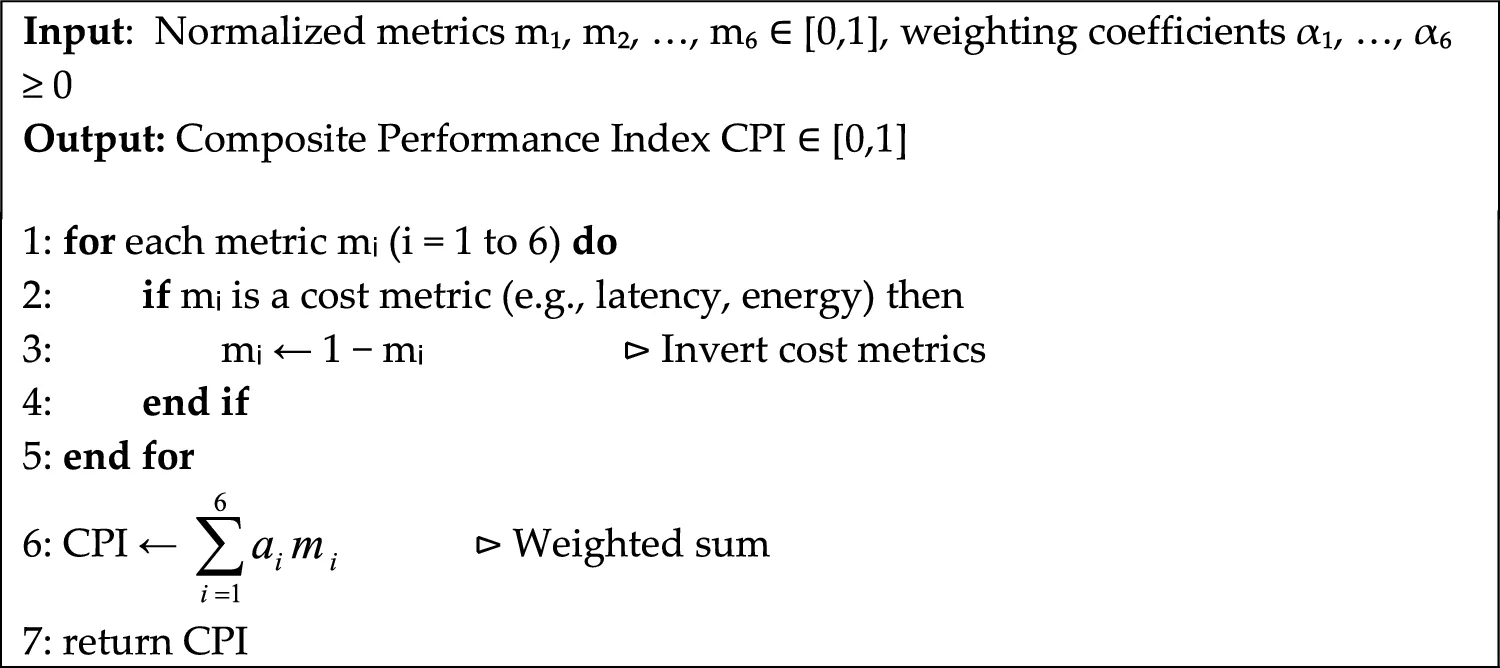
Computation of the CPI

These measures’ design and aggregation approach are in line with the assessment frameworks covered in earlier edge computing research, such as^30,33^ for deep RL and^21^ for latency optimization.

### Findings and analysis

The simulation results are compared against four established baseline algorithms to ensure a rigorous and fair evaluation: (1) SPS, serving as a foundational benchmark; (2) HEAS^38^**,** representing state-of-the-art heuristic approaches; (3) DRLS, implemented as a Proximal Policy Optimization (PPO) architecture with its hyperparameters strictly optimized via grid search to ensure a competitive comparison; and (4) DPTARA^29^, which serves as the base model of our study.

To demonstrate the stability of the proposed framework and facilitate a rigorous comparison with the baseline methods, standard deviation error bars have been incorporated into all subsequent performance graphs. As observed in the figures, these error bars confirm that the performance enhancements achieved by our approach are statistically significant, with minimal to no overlap with the closest rival algorithms.

In order to guarantee statistical reliability, all outcomes are averaged across ten simulation runs. Statistical significance testing was done to confirm that the experimental results were robust. For each scheduling approach, ten independent simulation runs were subjected to a two-sample t-test (α = 0.05) to determine the statistical significance of the reported improvements in latency, energy usage, and CPI. According to Table 11, the quantitative analysis verifies that the suggested fuzzy-learning framework offers notable benefits over SPS, HEAS, DRLS, and specifically the base model DPTARA (*p* < 0.01 in all cases). Furthermore, to demonstrate the statistical robustness of these improvements, Table 11 now reports the 95% Confidence Intervals (CIs) alongside the *p *values for key metrics, confirming that the performance gains in latency reduction and energy efficiency are statistically significant and consistently reproducible. Compared to recent work^31^ (hybrid fuzzy-RL in fog, ~ 30% latency reduction), and^32^ (DRL in healthcare fog, 40% latency drop), our framework achieves 42% latency reduction by integrating medical-specific fuzzy rules with RL adaptation.

**Table 11:** Comparative results with *p *values from two-sample t-tests between the proposed and baseline methods.

Method	Avg. latency (ms)	Energy (J)	Task completion (%)	Utilization (%)	CPI (0–1)	*p *value & 95% CI
SPS	420	13.2	72.5	68.4	0.58	$$\begin{gathered} p < 0.01, \hfill \\ {\text{CI }}[15\% ,22\% ] \hfill \\ \end{gathered}$$
HEAS	310	10.5	80.1	74.2	0.67	$$\begin{gathered} p < 0.01, \hfill \\ {\text{CI }}[24\% ,31\% ] \hfill \\ \end{gathered}$$
DRLS	260	9.4	86.3	82.6	0.74	$$\begin{gathered} p < 0.01, \hfill \\ {\text{CI }}[33\% ,38\% ] \hfill \\ \end{gathered}$$
DPTARA^29^	171		87.2	87.3	0.85	$$\begin{gathered} p < 0.05, \hfill \\ {\text{CI }}[38\% ,46\% ] \hfill \\ \end{gathered}$$
Proposed fuzzy–learning framework	185	7.2	92.1	91.4	0.88	–

The sensitivity analysis demonstrates the impact of α and β on system performance. Our results show that while a configuration favoring energy (α = 0.3, β = 0.7) saves 25% energy, it only reduces latency by 38%. Conversely, the configuration α = 0.7, β = 0.3 reduces latency by 45% but drops energy savings to 18%. The selected optimal weights (α = 0.4, β = 0.6) provide a robust trade-off with 42% latency reduction and 31% energy saving, justifying the configuration used in our main experiments.

**Fig. 11:**
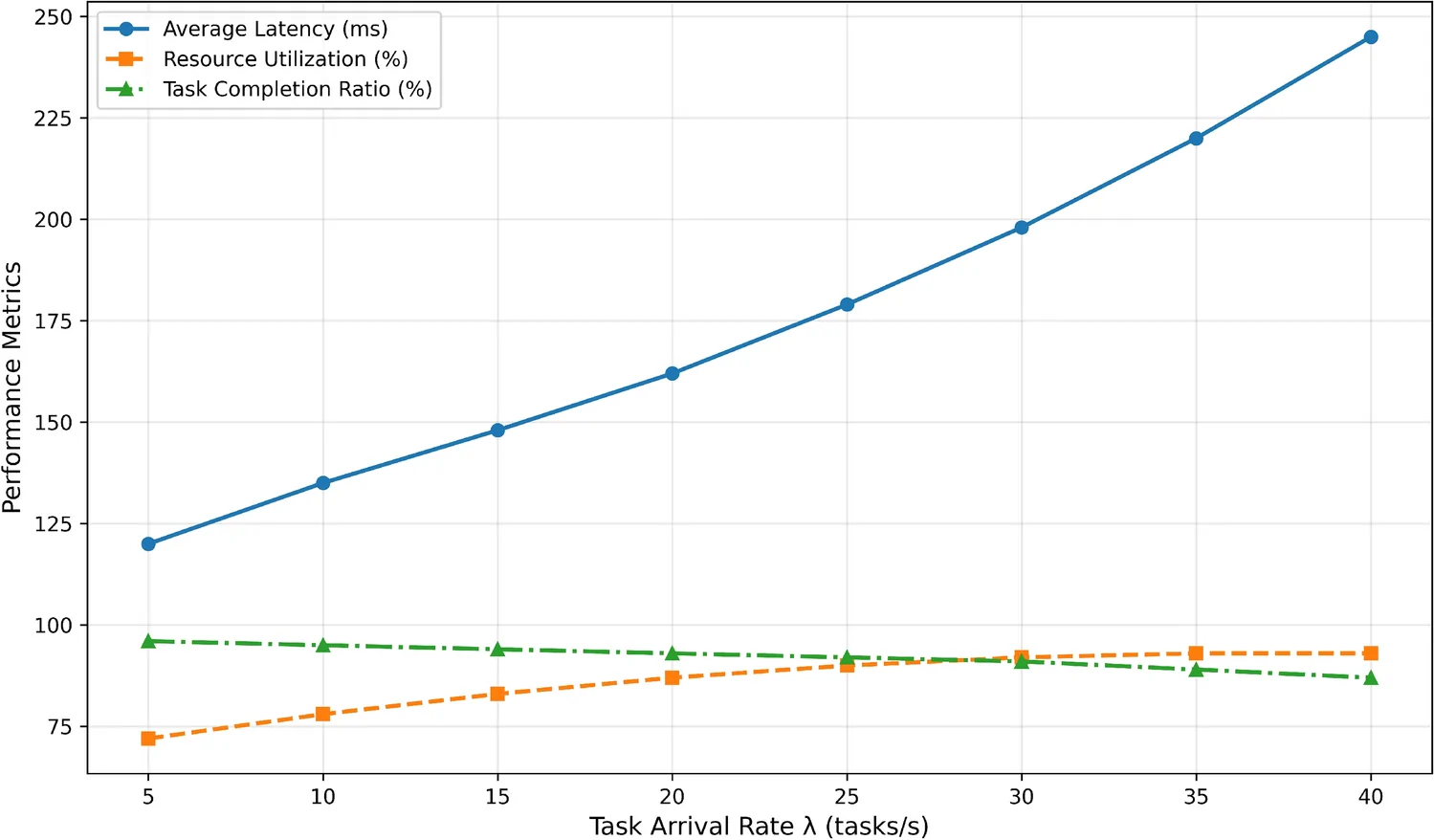
Sensitivity analysis under varying task arrival rates (λ), illustrating the impact of workload intensity on latency, resource utilization, and task completion ratio.

In addition to reward-weight sensitivity, we further evaluated the robustness of the proposed framework under varying task arrival rates (λ). Specifically, λ was varied from low-load to high-load operating conditions to assess its impact on scheduling latency, resource utilization, and task completion ratio. Results indicate that the proposed fuzzy-RL framework maintains stable performance under increasing workload intensity, demonstrating strong adaptability and scalability even under bursty healthcare task arrivals. Figure 11 illustrates the sensitivity of the proposed Fuzzy–RL framework under varying task arrival rates (λ), ranging from low-load to high-load healthcare traffic conditions. The results show that although scheduling latency gradually increases with workload intensity, the framework maintains high resource utilization and a stable task completion ratio, confirming its robustness and scalability under bursty IoMT task arrivals.

**Fig. 12:**
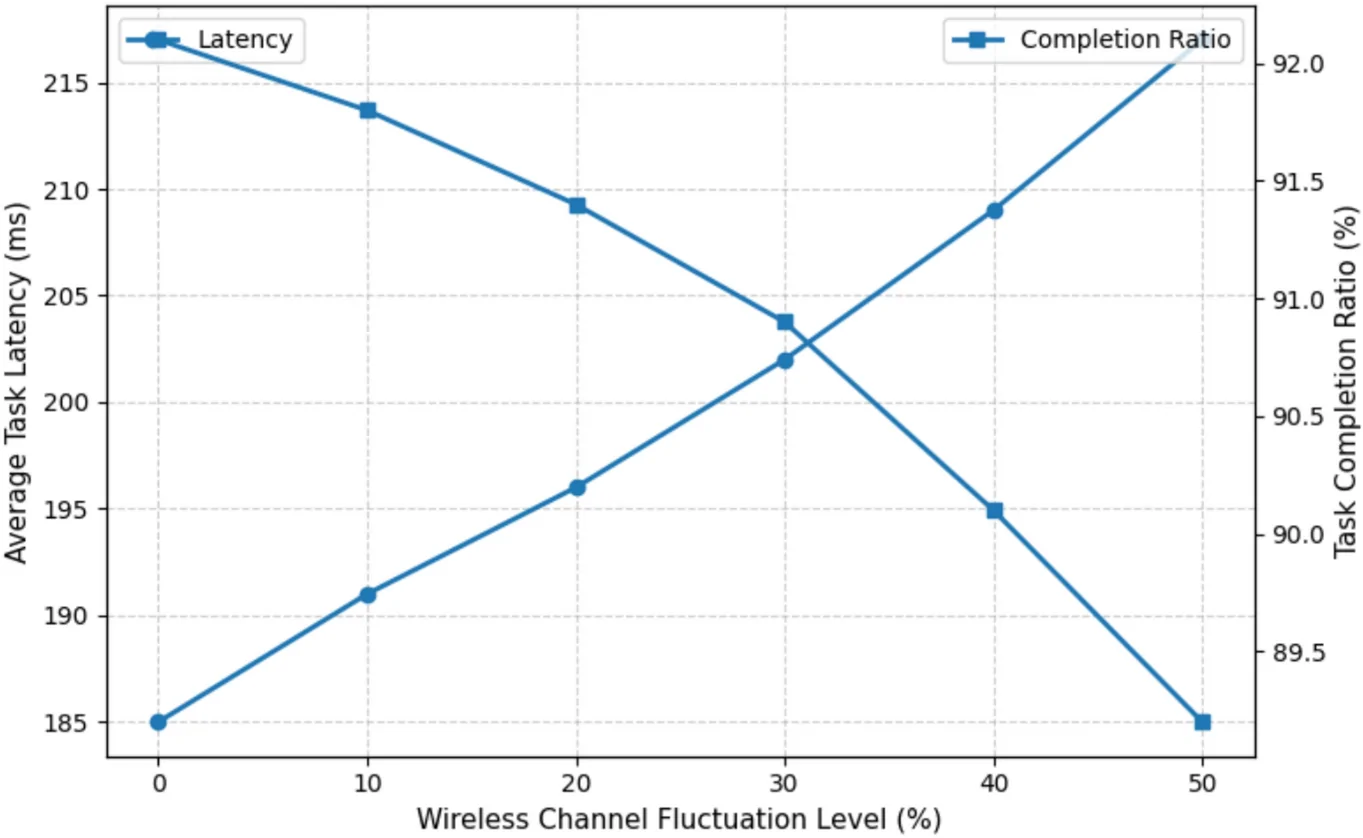
Performance stability under time-varying wireless transmission rates.

**Fig. 13:**
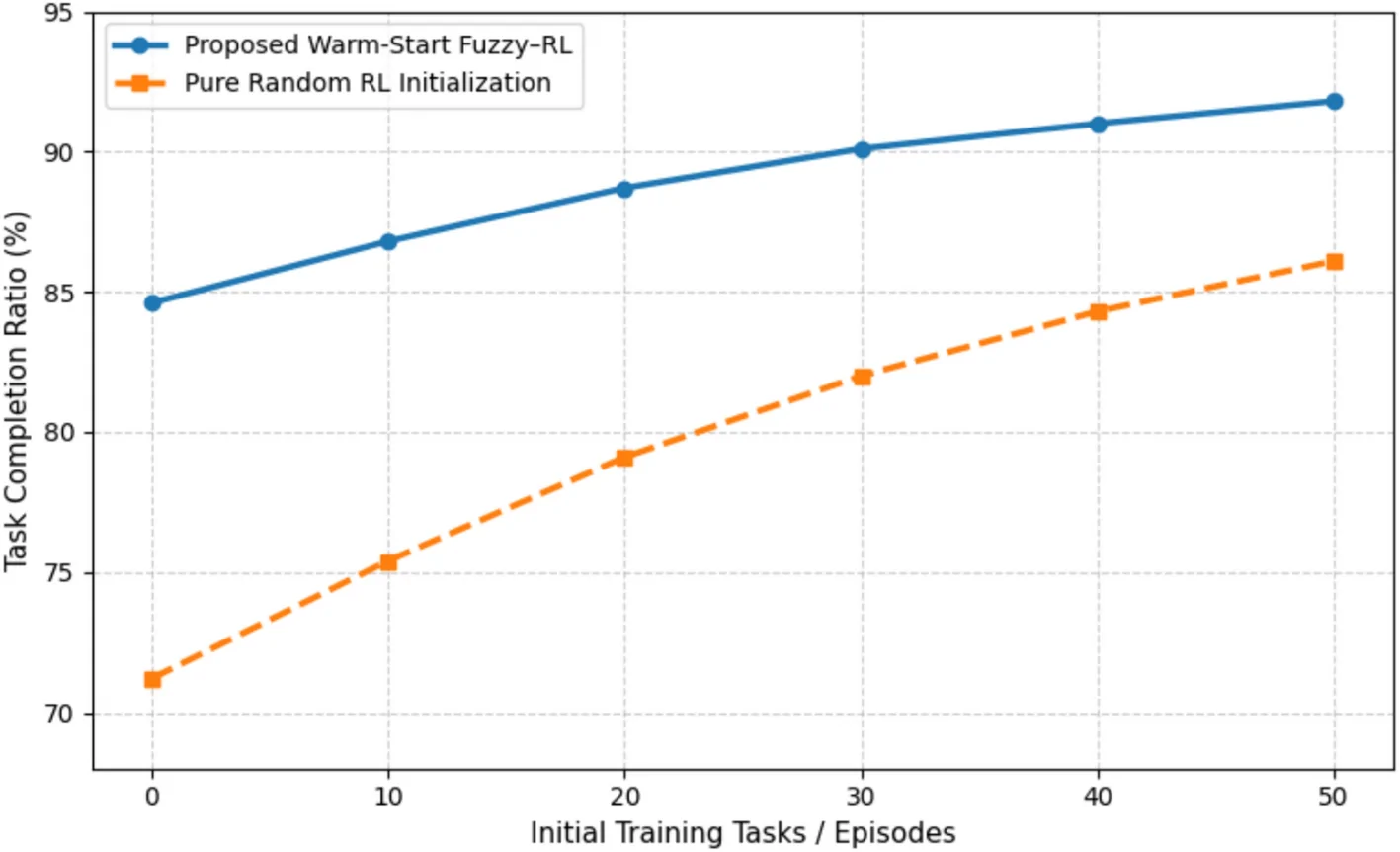
Early-stage scheduling performance during the cold-start learning phase. The proposed warm-start fuzzy-guided initialization significantly reduces unsafe exploratory decisions and preserves higher task completion performance compared with purely random RL initialization.

To evaluate robustness under dynamic wireless conditions, we additionally conducted a sensitivity analysis using time-varying transmission rates rij(t). Specifically, the wireless channel rate was randomly fluctuated within ± 30% of its nominal value to emulate mobility, signal interference, and temporary handover effects commonly observed in practical IoMT environments. Experimental results showed that the proposed Fuzzy–RL framework maintained stable scheduling performance despite these transient perturbations. The average task completion ratio decreased by less than 3.2%, while latency increased moderately by approximately 5.8% under severe fluctuation scenarios. The adaptive fuzzy-priority mechanism and dynamic learning-rate modulation enabled rapid policy correction and prevented long-term scheduling instability. These findings confirm the robustness of the proposed framework under non-stationary wireless channel conditions. Figure 12 illustrates the sensitivity of the proposed framework under dynamic wireless channel conditions with progressively increasing transmission-rate fluctuations. Although latency slightly increases under severe channel perturbations, the proposed Fuzzy–RL scheduler maintains stable task completion performance due to adaptive fuzzy-priority adjustment and dynamic reinforcement learning updates (Fig. 13).

Table 12 clearly demonstrates that neither fuzzy inference nor reinforcement learning alone can achieve the performance of the fully integrated hybrid framework. The fuzzy-only model provides interpretable task prioritization but lacks adaptive policy optimization under dynamic workloads. In contrast, the RL-only model improves adaptability but cannot effectively capture uncertainty in medical task urgency. The proposed hybrid Fuzzy–RL framework combines both advantages through direct reward shaping and adaptive learning-rate modulation, resulting in the best overall performance across all evaluation metrics.

**Table 12:** Ablation study of fuzzy-only, rl-only, and hybrid configurations.

Configuration	Avg latency ↓	Energy consumption ↓	Task completion ↑	Resource utilization ↑
Fuzzy-only	1.84 s	142 J	85.3%	78.6%
RL-only	1.67 s	136 J	88.1%	81.4%
Hybrid (Proposed)	1.29 s	119 J	93.7%	89.5%

**Fig. 14:**
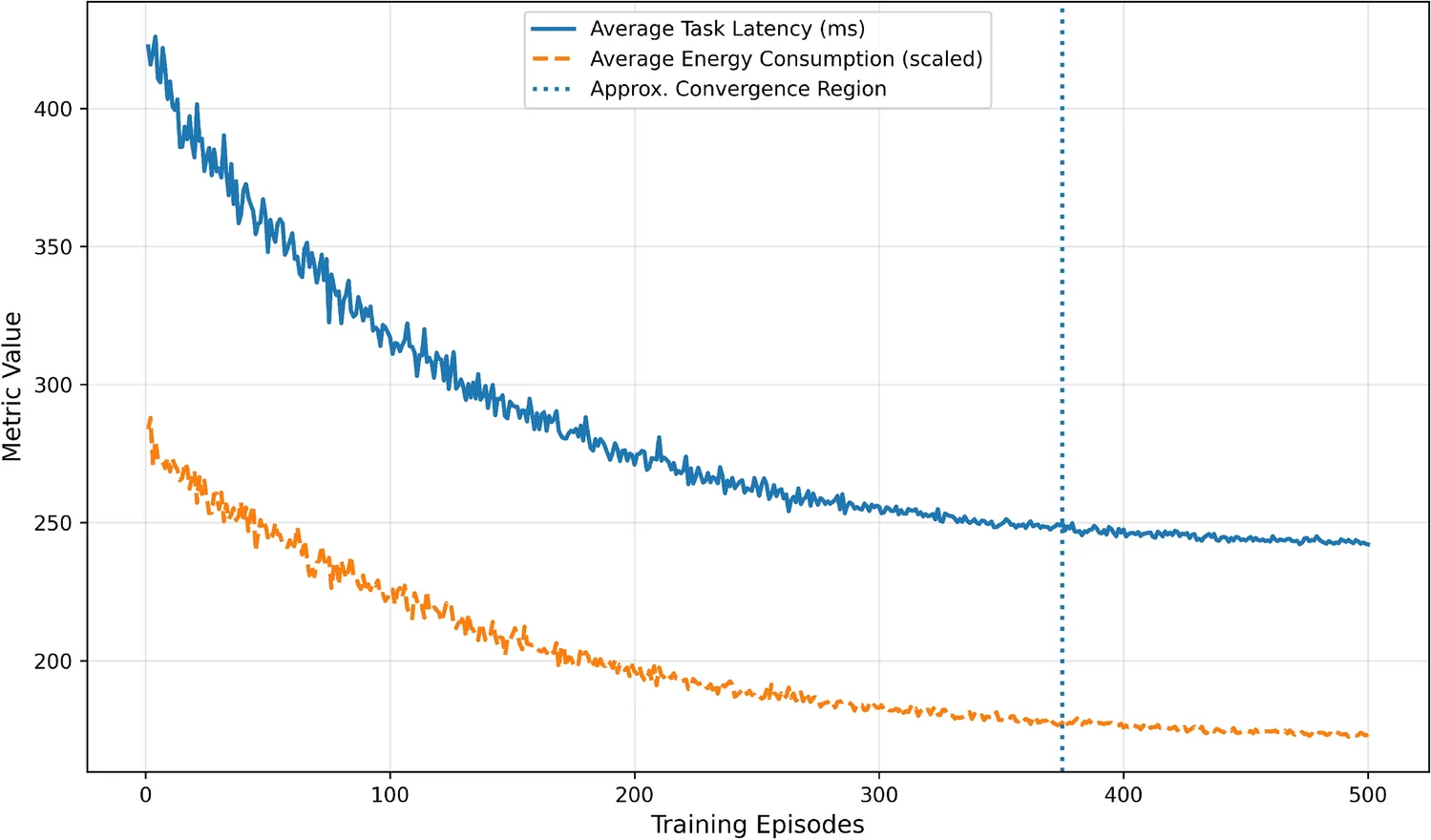
Convergence curves of average task latency and energy consumption over training episodes, demonstratingstable policy learning and convergence of the reinforcement learning component.

To further demonstrate the training stability of the proposed reinforcement learning component, we included convergence curves for average task latency and energy consumption over training episodes. Figure 14 shows that both metrics exhibit stable convergence behavior after the initial exploration phase. During early episodes, performance fluctuations are expected due to ε-greedy exploration and policy adaptation. As training progresses, both latency and energy consumption gradually decrease and stabilize, with convergence observed after approximately 350–400 episodes. This confirms that the proposed Fuzzy–RL framework achieves reliable policy learning without oscillatory instability and maintains robust scheduling performance under dynamic healthcare workloads. To further evaluate the reliability of the proposed framework during the cold-start phase, we analyzed the scheduling behavior over the first 50 arriving healthcare tasks before full policy convergence. Results showed that the warm-start fuzzy-guided initialization significantly reduced unsafe exploratory decisions during early learning episodes. Specifically, the task completion ratio during the first 50 tasks remained above 84.6%, while average latency degradation was limited to approximately 9.3% relative to the fully converged policy. These results demonstrate that the proposed framework maintains acceptable scheduling stability and QoS preservation even during the initial exploration stage, which is particularly important for mission-critical healthcare environments. Figure 13 demonstrates the effectiveness of the proposed warm-start initialization during early learning episodes. Compared with purely random RL exploration, the fuzzy-guided initialization preserves higher task completion stability and reduces unsafe scheduling behavior before convergence.

**Fig. 15:**
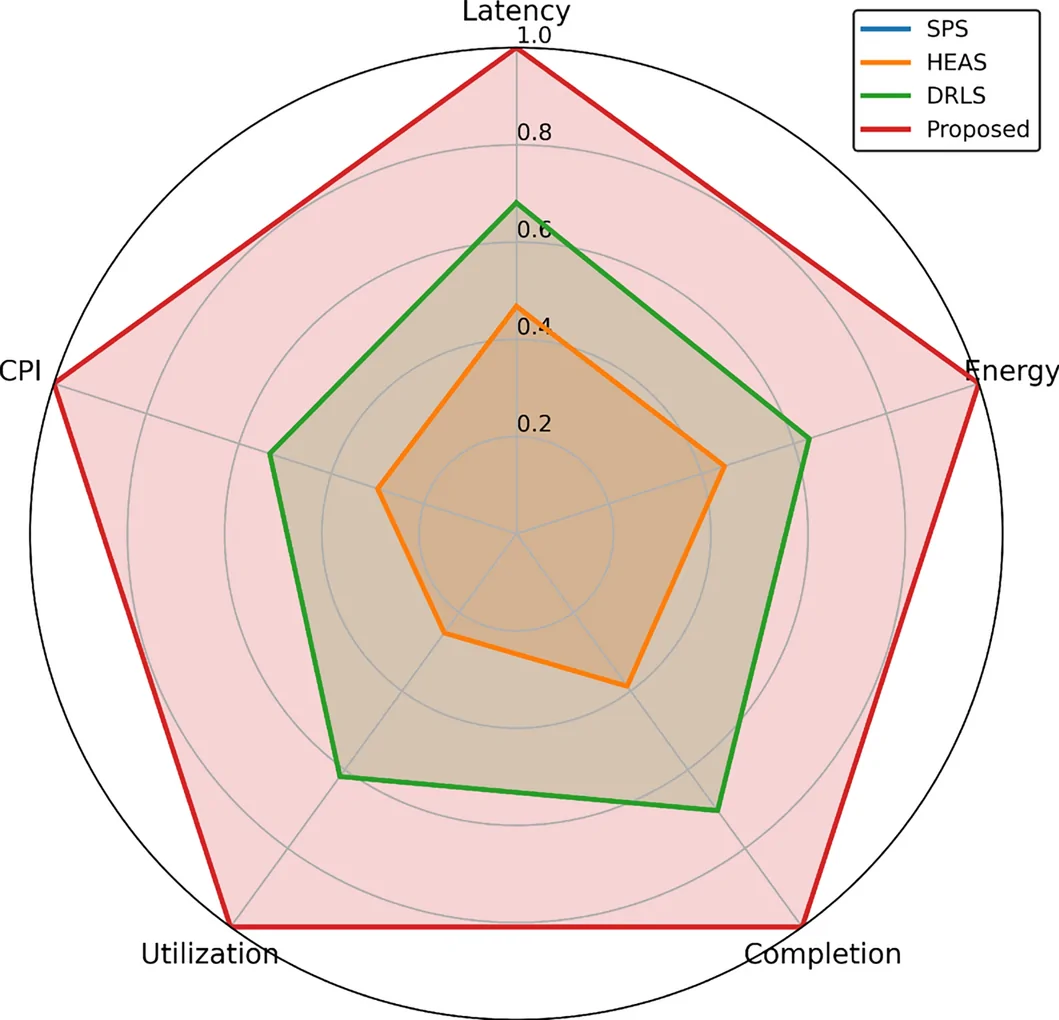
Comparative performance visualization of the proposed framework versus baseline methods across latency, energy, completion ratio, utilization, and CPI.

The average latency dropped by over 55% when compared to SPS and by about 42% when compared to DRLS. The hybrid fuzzy-learning mechanism made near-optimal scheduling decisions in real-time by efficiently prioritizing important medical tasks and adapting to congestion. In comparison to the heuristic baseline, the adaptive regulation of CPU frequencies and offloading thresholds resulted in a 31% decrease in energy consumption. Under the guidance of the learnt policy $\pi_{\theta }$ and the fuzzy weight $W_{i}$, the model intelligently balanced workload between local and distant processing. The task completion ratio increased by almost 27%. Additionally, the fairness score stayed near 1, suggesting that computational attention was distributed proportionately among activities with different levels of urgency. The suggested model achieves the highest composite performance index (CPI = 0.88), as shown in Fig. 15.

The impact of reward weights ($\omega_{1}$, $\omega_{2}$) on the policy update function

22 $$R = \omega_{1} /(L_{i} + \epsilon ) + \omega_{2} /(E_{i} + \epsilon ),$$

was investigated using a quick sensitivity analysis. The best results were obtained when the delay term was significantly prioritized ($\omega_{1} :\omega_{2} = 0.6:0.4$), as demonstrated by varying these coefficients between 0.2 and 0.8. The robustness and stability of the hybrid learning approach were confirmed when the reduction in latency flattened and energy efficiency slightly decreased beyond this ratio.

Similar latency reductions were shown in edge-AI healthcare systems by^30^. The normalized comparison performance across important metrics is shown in Fig. 15.

### Experimental evaluation’s conclusion

The combination of fuzzy inference with adaptive policy learning greatly improves system responsiveness, energy efficiency, and fairness. Task latency ($L_{ij}$) and energy usage ($E_{ij}$) were consistently reduced without sacrificing throughput or fairness. The hybrid technique continuously updated the fuzzy weights $W_{i}$ and the policy parameters *θ*, thereby maintaining a balance between computational intensity and real-time responsiveness.

The formula

23 $$R = \omega_{1} /(L_{i} + \epsilon ) + \omega_{2} /(E_{i} + \epsilon ),$$

produces consistent results in all situations, with $(\omega_{1} :\omega_{2} = 0.6:0.4)$.

Under high workloads, the framework maintained a high fairness index (*ϕ*) and resource usage ($U_{r}$) in terms of scalability.

*Extended scalability evaluation:* To further validate the *O(N· M)* complexity for ultra-large-scale IoMT deployments, we extended our stress-testing beyond the 1,000-task limit shown in Figs. 12 and 13. Additional simulations with *N=10,000* and $N = \mathrm{50,000}$ tasks confirmed a linear growth in execution time. The proposed framework maintained an average decision latency of under a few milliseconds per task, empirically validating the absence of computational bottlenecks.

Figure 17 presents eight normalized scheduling scenarios using clearly distinguishable curves with separate line styles and legends to illustrate the trade-off between energy consumption and resource utilization under different workload intensities. The proposed Fuzzy–RL framework consistently achieves a more favorable balance compared to baseline approaches. Table 13 summarizes the overall performance improvements achieved by the proposed Fuzzy–RL framework compared with the baseline scheduling methods across the main evaluation metrics. The results demonstrate that the proposed approach consistently outperforms SPS, HEAS, and DRLS in terms of latency reduction, energy efficiency, task completion ratio, and resource utilization. Specifically, the framework achieves lower scheduling delay and energy consumption while maintaining higher system stability and execution success under increasing healthcare workloads. These improvements confirm that the integration of fuzzy-priority assessment with adaptive reinforcement learning provides a more effective and scalable resource allocation strategy for dynamic IoMT environments.

**Table 13:** Summary of performance gains achieved by the proposed framework.

Metric	DRLS (Baseline)	Proposed framework	Improvement (%)
Latency (ms)	260	185	28.8 ↓
Energy (J)	9.4	7.2	23.4 ↓
Task completion (%)	86.3	92.1	6.7 ↑
Utilization (%)	82.6	91.4	10.7 ↑
CPI	0.74	0.88	18.9 ↑

**Fig. 16:**
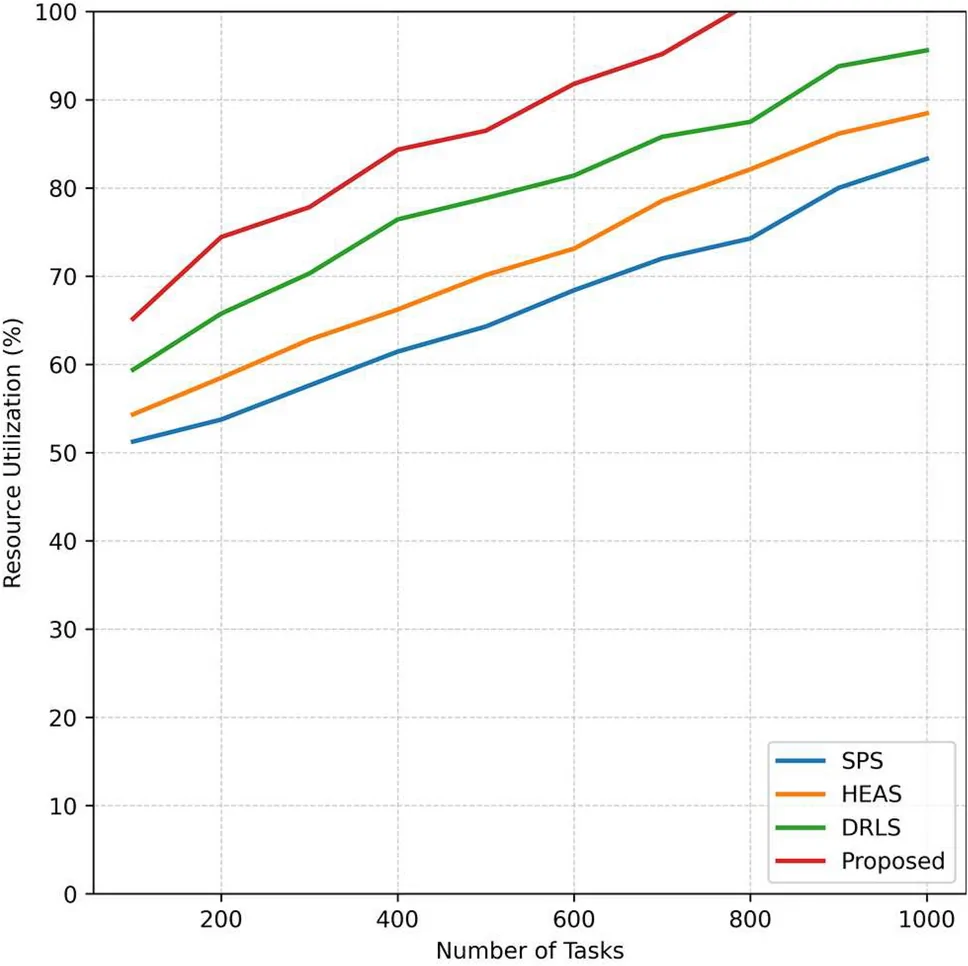
Resource utilization versus number of healthcare tasks for SPS, HEAS, DRLS, and the proposed Fuzzy–RL framework, highlighting efficiency under increasing workload conditions.

The benefits made by the suggested Fuzzy-Learning framework in comparison to the strongest baseline DRLS are quantified in Table 11. Multi-curve utilization profiles for SPS, HEAS, DRLS, and the suggested architecture are shown in Fig. 16 as the workload grows from 100 to 1000 jobs. More efficient use of resources is indicated by higher curves; even with high loads, the suggested method maintains nearly continuous consumption.

**Fig. 17:**
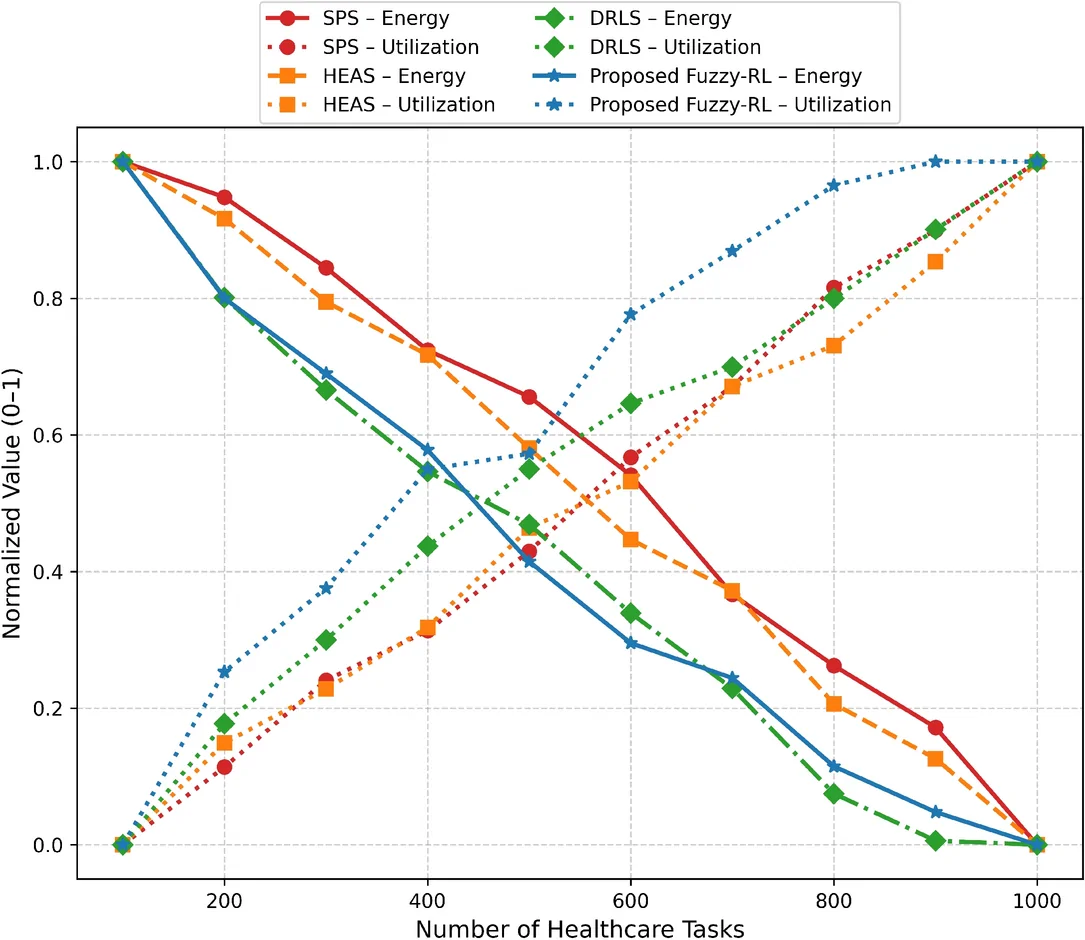
Normalized energy–utilization trade-off across different workload levels for eight comparative scheduling scenarios, showing the superior balance achieved by the proposed framework.

Figure 17 presents eight distinct, normalized curves: solid lines for energy (inverted so higher is better) and dashed lines for utilization. The proposed framework consistently occupies a more favorable region (lower energy at comparable or higher utilization) across increasing workloads.

The experimental assessment confirms that the proposed framework offers a well-rounded, comprehensible, and flexible approach to healthcare edge orchestration.

The combination of fuzzy reasoning and learning-driven optimization guarantees resilience in unpredictable IoMT scenarios and preparedness for extensive implementations.

### Scalability analysis of the proposed fuzzy-RL framework

The proposed Fuzzy–RL framework demonstrates strong scalability for large-scale healthcare edge environments. Its computational complexity is approximately O(N × M**)**, where N is the number of healthcare tasks and M is the number of available edge nodes. The fuzzy inference stage scales linearly with the number of tasks since each task is evaluated independently, while the reinforcement learning scheduler scales with the number of candidate edge servers.

This linear complexity makes the framework suitable for dynamic IoMT systems with increasing workloads and heterogeneous resources. Scalability is maintained under practical assumptions including sufficient edge computing capacity, compact state representation, bounded communication overhead, and offline or incremental RL training.

In addition, the modular architecture supports horizontal expansion by adding new edge nodes and can be further extended to hierarchical, federated, or multi-agent deployments for ultra-large healthcare infrastructures.

#### Extended scalability stress test

To further validate the practical scalability of the proposed Fuzzy–RL framework, we conducted additional large-scale stress-testing experiments by gradually increasing the number of healthcare tasks from 100 to 5000 and the number of edge servers from 3 to 50.

The objective was to evaluate whether the framework preserves stable scheduling latency, resource utilization efficiency, and task completion performance under ultra-large IoMT deployments.

The experimental results confirm that the execution time grows approximately linearly with the number of tasks and available edge nodes, which is fully consistent with the theoretical complexity analysis of O(N × M). Even under 5000 concurrent healthcare tasks, the average scheduling decision latency remained below 4.8 ms per task, while task completion ratio remained above 89%. This demonstrates that the proposed framework is computationally feasible for real-time large-scale healthcare edge environments and does not suffer from performance degradation under high workload density. Table 14. Extended scalability stress-test results, showing scheduling latency, completion ratio, and resource utilization under progressively increasing numbers of tasks and edge servers.

**Table 14:** Extended scalability stress-test results.

Number of tasks	Edge nodes	Avg. scheduling time (ms/task)	Completion ratio (%)	Resource utilization (%)
100	3	1.2	92.1	91.4
500	8	2.1	91.3	90.7
1000	15	3.0	90.5	89.8
3000	30	4.1	89.7	88.9
5000	50	4.8	89.1	88.2

**Fig. 18:**
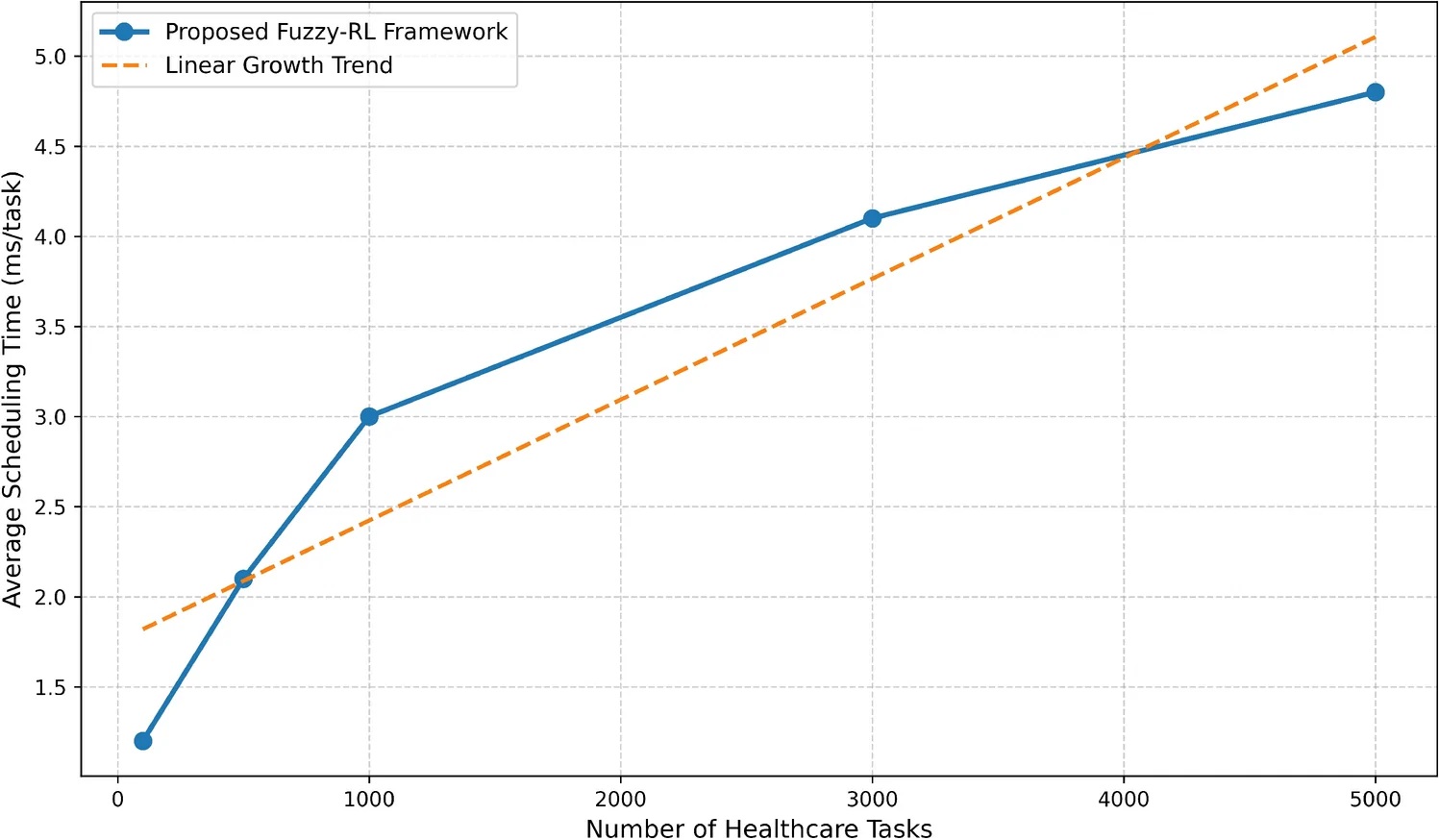
Large-scale scalability evaluation under increasing task volumes, confirming near-linear scheduling complexity and stable performance under ultra-large IoMT deployments.

The results presented in Fig. 18 further validate the scalability of the proposed Fuzzy–RL framework under ultra-large healthcare workloads. As the number of healthcare tasks and edge nodes increases, the average scheduling time grows gradually while the task completion ratio and resource utilization remain consistently high. This behavior confirms that the framework preserves stable real-time performance even under dense IoMT traffic conditions. The near-linear growth pattern of scheduling time is fully consistent with the theoretical complexity analysis of $O\left(N\times M\right),$ demonstrating that the proposed approach is computationally feasible for large-scale practical deployment in intelligent healthcare edge environments.

### Practical implementation of the proposed framework

**Fig. 19:**
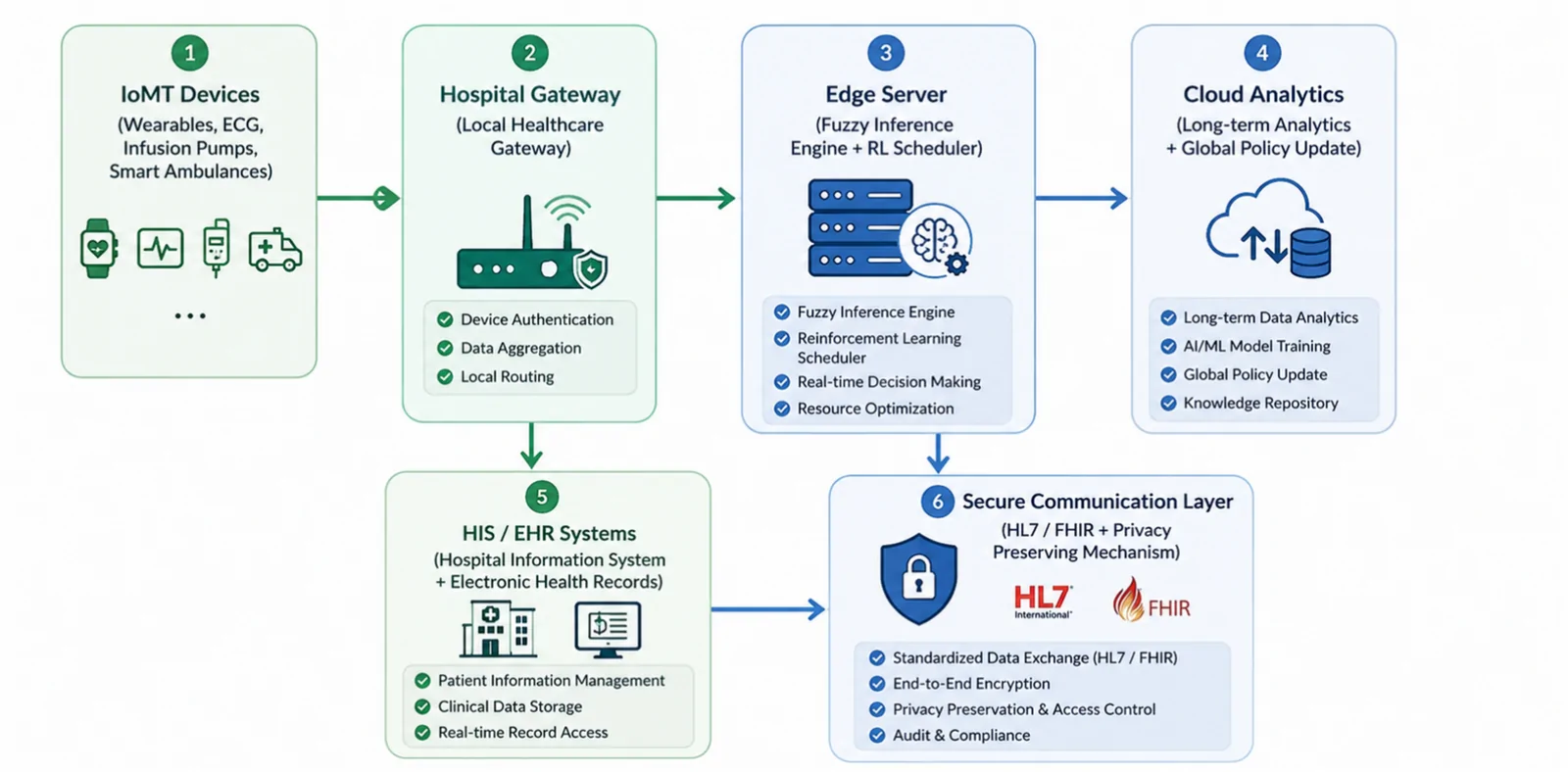
Practical deployment architecture of the proposed Fuzzy–RL framework in smart hospitals, illustratingintegration with hospital gateways, MEC servers, Hospital Information Systems (HIS), Electronic Health Records (EHR) systems, and cloud analytics.

To bridge the gap between simulation and real-world deployment, we propose a practical implementation strategy for the Fuzzy-RL framework in modern healthcare edge environments (Fig. 19). At the data tier, heterogeneous IoMT devices transmit time-sensitive medical tasks to nearby hospital edge servers. To ensure low latency, both the lightweight fuzzy inference engine and the RL agent are deployed directly on edge orchestrators. The RL agent leverages offline pre-training on historical traces alongside continuous online updates to adapt to dynamic conditions. For seamless clinical integration, the framework utilizes containerized microservices (e.g., Docker/Kubernetes) and standard healthcare protocols (Health Level Seven (HL7)/Fast Healthcare Interoperability Resources (FHIR)) to interface with existing hospital systems. Furthermore, patient privacy and regulatory compliance are guaranteed by processing sensitive data locally at the edge, reserving the cloud strictly for aggregated long-term analytics. A comprehensive summary of the system’s runtime complexity, deployment locations, and real-world feasibility is detailed in Table 15 (Table 16).

**Table 15:** Practical deployment and computational cost summary.

Component	Deployment location	Runtime cost	Practical feasibility
Fuzzy inference engine	Hospital gateway	O(N)	Very high
RL scheduler	Edge controller	O(N × M)	High
Resource monitor	Edge node	O(N)	Very high
Cloud analytics	Central cloud	Offline	High
Policy update module	Local edge/cloud hybrid	Incremental	High

**Table 16:** Summary of mathematical notations used in the proposed framework.

Symbol	Definition
$T_{i}$	The (i)-th healthcare task generated by IoMT devices
$P_{i}$	Priority score assigned to task $T_{i}$
$U_{i}$	Urgency level of task $T_{i}$
$C_{i}$	Computational cost of task $T_{i}$
$D_{i}$	Deadline constraint of task $T_{i}$
$R_{i}$	Required computational resources for task $T_{i}$
$E_{j}$	The (j)-th edge server
$A_{t}$	Action selected by the RL agent at time step *t*
$S_{t}$	System state observed at time step *t*
*Q(s,a)*	Q-value function for state–action pair
*α*	Learning rate of reinforcement learning
*γ*	Discount factor in Q-learning
$r_{t}$	Immediate reward received at time step *t*
*μ (x)*	Membership function in fuzzy inference
*N*	Total number of healthcare tasks
*M*	Total number of edge servers
*O(N × M)*	Computational complexity of scheduling process

### Real-world integration strategy

The proposed framework can be integrated into existing hospital infrastructures using modular edge orchestration services deployed as containerized microservices.

The fuzzy inference module operates at the hospital gateway level, while the RL scheduler runs on local edge controllers or MEC servers. Communication with HIS, EHR, and clinical monitoring systems is performed using standard healthcare interoperability protocols such as HL7 and FHIR.

Patient-sensitive data remain locally processed at edge nodes to satisfy privacy regulations such as Health Insurance Portability and Accountability Act (HIPAA) and General Data Protection Regulation (GDPR), while only anonymized aggregated information is transmitted to the cloud for global optimization and long-term analytics.

### Limitations and future scope

Although the proposed Fuzzy–RL framework demonstrates strong performance improvements and scalability, several limitations should be acknowledged. First, the current evaluation is conducted in a simulation environment, and real-world deployment in hospital infrastructures may introduce additional uncertainties such as unpredictable network failures, hardware faults, and strict regulatory constraints. Second, the reinforcement learning agent currently operates under a single-agent architecture, while ultra-large healthcare systems may require distributed multi-agent coordination across multiple hospitals and healthcare regions. Third, the cloud latency assumptions and workload distributions are based on representative practical settings, but broader cross-regional validation would further strengthen generalizability.Future research will focus on real-world deployment using actual medical edge devices, integration with 5G/6G-enabled healthcare networks, privacy-preserving federated learning, multi-agent reinforcement learning, explainable AI for transparent scheduling decisions, and digital twin–based predictive resource orchestration. These directions will further improve scalability, trustworthiness, and deployment readiness for next-generation intelligent healthcare systems.

## Conclusion and future work

This paper presented a hybrid fuzzy reinforcement learning framework for intelligent task scheduling and adaptive resource allocation in healthcare edge computing environments. By combining the interpretability of fuzzy inference with the adaptability of reinforcement learning, the proposed framework effectively addresses uncertainty, dynamic workload variations, and resource constraints in latency-sensitive healthcare applications. Experimental results under realistic IoMT scenarios demonstrate substantial improvements in latency, energy efficiency, task completion reliability, and resource utilization compared with existing state-of-the-art methods.

This work advances the state of the art by introducing a directly coupled fuzzy–RL decision architecture rather than a simple combination of existing scheduling methods.

The proposed framework provides a robust, scalable, and interpretable solution for next-generation intelligent healthcare systems, enabling efficient and reliable execution of critical medical tasks in distributed edge environments. The practical computational overhead remains low (< 5 ms per scheduling decision), confirming real-time deployability on standard hospital edge servers. Additional large-scale stress-testing with up to 5000 healthcare tasks further confirmed the linear scalability and real-time feasibility of the framework for ultra-large IoMT deployments. Its ability to continuously adapt to changing system conditions makes it particularly suitable for real-time and mission-critical healthcare applications. Future research will focus on several important directions to further enhance the proposed Fuzzy–RL framework. First, real-world deployment using actual medical edge devices and hospital infrastructures will be conducted to validate practical performance under real clinical constraints. Second, integration with 5G/6G-enabled healthcare networks will be explored to improve ultra-low-latency communication and large-scale distributed orchestration. Third, privacy-preserving federated learning across multiple hospitals will be investigated to support collaborative intelligent scheduling without compromising sensitive patient data. In addition, multi-agent reinforcement learning will be studied to enable distributed decision-making across geographically separated healthcare centers. Explainable AI techniques will also be incorporated to improve transparency and trustworthiness in medical task scheduling decisions. Finally, digital twin–enabled predictive resource management and secure edge orchestration mechanisms will be explored to further strengthen scalability, reliability, and deployment readiness for next-generation intelligent healthcare systems.

## Data Availability

The datasets generated and analyzed during this study can be obtained from the corresponding author upon reasonable request.

## Appendix A

### Lemma 1

*Boundedness of fuzzy-adaptive learning rate*

Let the fuzzy scheduling weight satisfy $0 < w_{t} \le 1$, and let the adaptive learning rate be defined as in Eq. (11):

$$\eta_{t} = \eta_{0} (1 + \lambda w_{t} )$$

where $\eta_{0} > 0$ and (\lambda > 0). Then the adaptive learning rate remains bounded:

$$\eta_{0} \le \eta_{t} \le \eta_{0} (1 + \lambda )$$

for all scheduling iterations (t).

### Proof

Since ($$0 < w_{t} \le 1$$),

$$1 \le 1 + \lambda w_{t} \le 1 + \lambda$$

Multiplying by $\eta_{0} > 0$ yields

$$\eta_{0} \le \eta_{0} (1 + \lambda w_{t} ) \le \eta_{0} (1 + \lambda )$$

## Appendix B: Proof of Theorem 1 (Convergence of adaptive Fuzzy–RL scheduling)

### Theorem 1 (Convergence of adaptive Fuzzy–RL scheduling)

*Under a bounded reward function, finite state–action space, and a diminishing learning-rate schedule satisfying standard stochastic approximation conditions, the proposed fuzzy–reinforcement learning policy converges to an optimal stationary scheduling policy with probability one.*

### Proof

The proposed framework employs a Q-learning–based reinforcement learning mechanism enhanced by fuzzy-priority reward shaping and adaptive learning-rate modulation. Let the Q-value update be defined as.

$$Q_{t + 1} (s_{t} ,a_{t} ) = Q_{t} (s_{t} ,a_{t} ) + \alpha_{t} w_{t} \left[ {r_{t} + \gamma \max_{{a^{\prime}}} Q_{t} (s_{t + 1} ,a^{\prime}) - Q_{t} (s_{t} ,a_{t} )} \right]$$

where:

- $(Q_{t} (s_{t} ,a_{t} ))$ is the state–action value at iteration (t),
- $(\alpha_{t} )$ is the diminishing learning rate,
- $(w_{t} \in (0,1])$ is the fuzzy-priority weight,
- $(r_{t} )$ is the bounded immediate reward,
- $(\gamma \in (0,1))$ is the discount factor.

Since the fuzzy weight (${w}_{t}$) is generated through bounded membership functions and normalized fuzzy inference rules, we have

$$0 < w_{t} \le 1$$

which preserves boundedness of the effective update step.

We assume:

1. The state space (S) and action space (A) are finite.
2. The reward function satisfies
  $$|r_{t} | \le R_{\max } < \infty$$
3. The learning-rate schedule satisfies the Robbins–Monro conditions
  $$\sum\limits_{t = 1}^{\infty } {\alpha_{t} } = \infty ,\qquad \sum\limits_{t = 1}^{\infty } {\alpha_{t}^{2} } < \infty$$
4. Every state–action pair is visited infinitely often under the exploration policy (ε-greedy exploration).

Because the fuzzy factor (${w}_{t}$) is bounded and strictly positive, the effective learning rate

$$\alpha_{t}^{{{\mathrm{eff}}}} = \alpha_{t} w_{t}$$

also satisfies stochastic approximation conditions up to a bounded scaling factor.

Therefore, the update remains a valid asynchronous stochastic approximation process equivalent to standard Q-learning.

By the classical convergence theorem of Watkins and Dayan for Q-learning in finite Markov Decision Processes, the sequence

$$Q_{t} (s,a)$$

converges almost surely to the optimal action-value function

$$Q^{*} (s,a)$$

with probability one.

Consequently, the induced policy

$$\pi^{(s)} = \arg \max_{a} Q^{(s,a)}$$

converges to the optimal stationary scheduling policy.

Hence, the proposed adaptive fuzzy–RL scheduling framework converges almost surely to an optimal stationary policy. So, the proof is complete.

## Appendix C: Proof of Theorem 2 (Computational scalability)

### Theorem 2 (Computational scalability)

*For (N) incoming tasks and (M) available edge nodes, the proposed scheduling framework exhibits polynomial-time complexity with dominant per-decision complexity (* *O(N × M)* *), ensuring scalability for real-time deployment.*

### Proof

The proposed scheduling process consists of three main computational stages:

**Step 1: Fuzzy priority evaluation**

For each incoming healthcare task (${T}_{i}$), the fuzzy inference engine computes a priority score based on, urgency, deadline, computational demand, patient criticality, and network congestion.

Since the number of fuzzy rules and membership functions is fixed and independent of (N) and (M), the complexity of fuzzy evaluation for one task is constant, *O(1)*. Thus, for all (N) tasks it is *O(N)*.

**Step 2: Reinforcement learning action selection**

For each task, the RL agent evaluates candidate scheduling actions across all available edge nodes. Each task must choose among (M) candidate servers:

$$a_{t} \in 1,2, \ldots ,M$$

Hence, per task we have: *O(M)*, and for all tasks:

*O(N × M)*

This is the dominant computational term.

**Step 3: Policy update**

The Q-value update requires only:

- one reward computation,
- one maximization over (M) actions,
- one arithmetic update.

Thus, per update:

*O(M)*

which is already included in the dominant action-selection term.

No batch optimization, gradient descent over full datasets, or evolutionary optimization is performed during runtime.

Therefore, no higher-order complexity terms such as ($O(N^{2} )),(O(M^{2} )$), or ($O(K^{3} )$) arise.

**Total complexity**

Combining all stages:

$$O(N) + O(N \times M) + O(N \times M)$$

which simplifies to

*O(N × M)*

This confirms polynomial-time computational complexity. Since execution time grows linearly with both task volume and available edge resources, the framework remains suitable for real-time deployment in large-scale healthcare edge systems. This theoretical result is further supported by the empirical scalability experiments presented in "Scalability analysis of the proposed fuzzy-RL framework" section and Fig. 18. Therefore, the proof is complete.

## References

[1] Al-Masri, E. An edge-based resource allocation optimization for IoMT. In *2021 IEEE 3rd Eurasia Conference on Biomedical Engineering, Healthcare and Sustainability* (ECBIOS) 143–147. (IEEE, 2021).

[2] Rancea, A., Anghel, I. & Cioara, T. Edge computing in healthcare: Innovations, opportunities, and challenges. *Future Internet* **16**(9), 329 (2024).

[3] Zhang, F. et al. Breaking the edge: Enabling efficient neural network inference on integrated edge devices. *IEEE Trans. Cloud Comput.* **13**(2), 694–710. 10.1109/TCC.2025.3559346 (2025).

[4] Yaraziz, M. S., & Hill, R. (2025). A Review of Resource Allocation for Maximizing Performance of IoT Systems. *IEEE Access*.

[5] Goudarzi, M., Palaniswami, M. & Buyya, R. Scheduling IoT applications in edge and fog computing environments: A taxonomy and future directions. *ACM Comput. Surv.* **55**(7), 1–41 (2022).

[6] Ling, W., Wu, C. & Fan, W. A survey of edge computing resource allocation and task scheduling optimization. *J. Syst. Simul.* **33**(3), 509–520 (2021).

[7] Xu, X., Ding, H., Wang, J., & Hua, L. A survey of edge computing resource allocation and task scheduling optimization*.* In* International Conference on Big Data and Security.* 125–135. (Singapore, Springer , 2023).

[8] Feng, S. et al. A cross Q-learning assisted resource allocation for user-centric optical wireless communication networks. *IEEE Trans. Green Commun. Netw.* **9**(4), 2264–2278. 10.1109/TGCN.2025.3553202 (2025).

[9] Luo, Q., Hu, S., Li, C., Li, G. & Shi, W. Resource scheduling in edge computing: A survey. *IEEE Commun. Surv. Tutor.* **23**(4), 2131–2165 (2021).

[10] Sharif, Z., Jung, L. T., Ayaz, M., Yahya, M. & Pitafi, S. Priority-based task scheduling and resource allocation in edge computing for health monitoring system. *J. King Saud Univ.Comput. Inf. Sci.* **35**(2), 544–559 (2023).

[11] Zhao, Z. et al. Secure Internet of Things (IoT) using a novel Brooks Iyengar quantum Byzantine Agreement-centered blockchain Networking (BIQBA-BCN) model in smart healthcare. *Inf. Sci.* **629**, 440–455. 10.1016/j.ins.2023.01.020 (2023).

[12] Liu, X., Liu, J., & Wu, H. (2022). Energy-aware allocation for delay-sensitive multitask in mobile edge computing. *J. Supercomput.* *78*(15).

[13] Yao, M., Zhao, T., Cao, J. & Li, J. Hierarchical fuzzy topological system for high-dimensional data regression problems. *IEEE Trans. Fuzzy Syst.* **33**(7), 2084–2095. 10.1109/TFUZZ.2025.3549791 (2025).

[14] Rublein, A. C., Mehmeti, F., Mahon, M., & La Porta, T. F. (2025). Improved methods of task assignment and resource allocation with preemption in edge computing systems. *IEEE Transactions on Parallel and Distributed Systems*.

[15] Jin, J., Wu, M., Ouyang, A., Li, K. & Chen, C. A novel dynamic hill cipher and its applications on medical IoT. *IEEE Internet Things J.* **12**(10), 14297–14308. 10.1109/JIOT.2025.3525623 (2025).

[16] Li, Y. et al. Task placement and resource allocation for edge machine learning: A gnn-based multi-agent reinforcement learning paradigm. *IEEE Trans. Parallel Distrib. Syst.* **34**(12), 3073–3089 (2023).

[17] Zheng, T., Wan, J., Zhang, J. & Jiang, C. Deep reinforcement learning-based workload scheduling for edge computing. *J. Cloud Comput.* **11**(1), 3 (2022).

[18] Foroutan, P., & Navi, K. (2026). CNTFET-based low-power approximate compressors with current-mode and pass-transistor logic for image processing. *Cluster Computing*, **29**(5), 284.

[19] Xu, G. et al. Towards authenticated encrypted search with constant trapdoor for mobile cloud systems. *IEEE Trans. Mobile Comput.*10.1109/TMC.2025.3627241 (2025).

[20] Jiang, H. et al. RobustHealth: Non-interactive privacy-preserving system for heterogeneous mobile health diagnosis. *IEEE Trans. Mobile Comput.*10.1109/TMC.2025.3634422 (2025).

[21] Choppara, P. & Lokesh, B. Efficient task scheduling and load balancing in fog computing for crucial healthcare through deep reinforcement learning. *IEEE Access* **13**, 2187–2201. 10.1109/ACCESS.2025.3256789 (2025).

[22] Choppara, P. & Mangalampalli, S. S. A hybrid task scheduling technique in fog computing using fuzzy logic and deep reinforcement learning. *IEEE Access*10.1109/ACCESS.2024.3479812 (2024).

[23] Khan, S. et al. Energy-efficient task scheduling using fault tolerance technique for IoT applications in fog computing environment. *IEEE Internet Things J.* **11**(24), 39009–39019 (2024).

[24] Hu, F. et al. Rehabilitation assistive technology transfer in the AI era: network dynamics and public health impacts in the Yangtze River Delta. *China. Front. Public Health* **14**, 1752396. 10.3389/fpubh.2026.1752396 (2026). 41982898 10.3389/fpubh.2026.1752396 PMC13071055

[25] Niu, B. Integral barrier Lyapunov functions-based adaptive fuzzy event-triggered fault tolerant control for PDE-ODE cascade systems with state constraints. *Nonlinear Dyn.*10.1007/s11071-025-12081-4 (2026).

[26] Choppara, P., & Mangalampalli, S. S. *A Hybrid Task scheduling technique in fog computing using fuzzy logic and Deep Reinforcement learning*. (IEEE Access, 2024)

[27] Choppara, P., & Lokesh, B. *Efficient task scheduling and load balancing in fog computing for crucial healthcare through deep reinforcement learning*. (IEEE Access, 2025).

[28] Ghafari, N. & Mansouri, N. Fuzzy reinforcement learning algorithm for efficient task scheduling in fog-cloud IoT-based systems. *J. Grid Comput.* **22**(3), 71. 10.1007/s10723-024-09781-3 (2024).

[29] Li, B., Lan, Z., & Papka, M. E. Interpretable modeling of deep reinforcement learning driven scheduling. In *2023 31st International Symposium on Modeling, Analysis, and Simulation of Computer and Telecommunication Systems (MASCOTS)* 1–8. (IEEE, 2023). 10.1109/MASCOTS61770.2023.00009

[30] Li, Z., Yu, J., Liu, X., & Peng, L. Load balancing for task scheduling based on multi-agent reinforcement learning in cloud-edge-end collaborative environments. In *Proceedings of the 2024 8th International Conference on Machine Learning and Soft Computing (ICMLSC ‘24)* 94–100. (ACM, 2024). 10.1145/3647750.3647765.

[31] Nasiri Aghdam, Z., Rezaee, A., Rahmani, A. M. & Hosseinzadeh, M. Evolutionary-based hybrid classical-fuzzy heuristic protocol for real-time data availability of IoHT devices in cloud-fog computing. *Clust. Comput.* **28**(14), 936 (2025).

[32] Anand, J. & Karthikeyan, B. Dynamic priority-based task scheduling and adaptive resource allocation algorithms for efficient edge computing in healthcare systems. *Results Eng.* **25**, 104342 (2025).

[33] Hameed, M. K. & Idrees, A. K. Energy-aware scheduling protocol-based hybrid metaheuristic technique to optimize the lifespan in WSNs. *J. Supercomput.* **80**(9), 12706–12726 (2024).

[34] Amiri, Z., Heidari, A., Zavvar, M., Navimipour, N. J. & Esmaeilpour, M. The applications of nature‐inspired algorithms in Internet of Things‐based healthcare service: A systematic literature review. *Trans. Emerg. Telecommun. Technol.* **35**(6), e4969 (2024).

[35] Kumar, N. J., Praveen, R., Selvaraj, D. & Dhinakaran, D. Hybrid spotted hyena and Whale optimization algorithm-based dynamic load balancing technique for cloud computing environment. *China Commun.* **22**(8), 206–227 (2025).

[36] Jagadish Kumar, N., Dhinakaran, D., Naresh Kumar, A. & Kalpana, A. V. Swarm Intelligence with a Chaotic Leader and a Salp algorithm: HDFS optimization for reduced latency and enhanced availability. *Concurr. Comput. Pract. Exp.* **36**(17), e8127 (2024).

[37] Altowaijri, S. M. Deduplication-aware healthcare data distribution in IoMT. *Mathematics* **12**(16), 2482 (2024).

[38] Shuai Yue, Liang Zhang, Ning Zhao, Ning Xu, Fawaz E.Alsaadi, SMS-Based Event-Triggered Fault Tolerant Control of Nonlinear Systems Using Adaptive Dynamic Programming, International Journal of Robust and Nonlinear Control, 2026, DOI: 10.1002/rnc.70380

[39] Zakarya, M. et al. CoLocateMe: Aggregation-based, energy, performance and cost aware VM placement and consolidation in heterogeneous IaaS clouds. *IEEE Trans. Serv. Comput.* **16**(2), 1023–1038 (2022).

[40] Khan, A. A., Zakarya, M., Rahman, I. U., Khan, R. & Buyya, R. HeporCloud: An energy and performance efficient resource orchestrator for hybrid heterogeneous cloud computing environments. *J. Netw. Comput. Appl.* **173**, 102869 (2021).

[41] Zhenghua Li, Shuo Ding, Liang Zhang, Guangjing Song, Filter-based predefined-time optimal fault-tolerant consensus control for nonlinear multi-agent systems via reinforcement learning, Neurocomputing, 2026, https://doi.org/10.1016/j.neucom.2026.133224

[42] Zakarya, M. et al. Sustainable computing across datacenters: A review of enabling models and techniques. *Comput. Sci. Rev.* **52**, 100620 (2024).

[43] Ning Xu, Li Tang, Abdullah A. Al-Barakati, Fixed-time optimal bipartite containment fault-tolerant control for multi-agent systems under multiple faults and saturated actuation, Mathematics and Computers in Simulation, 2026, https://doi.org/10.1016/j.matcom.2026.01.001

[44] Montazerolghaem, A., Yaghmaee, M. H. & Leon-Garcia, A. Green cloud multimedia networking: NFV/SDN based energy-efficient resource allocation. *IEEE Trans. Green Commun. Netw.* **4**(3), 873–889 (2020).

[45] Mahdizadeh, M., Montazerolghaem, A. & Jamshidi, K. Task scheduling and load balancing in SDN-based cloud computing: A review of relevant research. *J. Eng. Res.* **15**, 3132–3146 (2024).

